# Neuropsychological Performance after Extended N-Pep-12 Dietary Supplementation in Supratentorial Ischemic Stroke

**DOI:** 10.3390/brainsci14100986

**Published:** 2024-09-28

**Authors:** Dafin Mureșanu, Olivia Verișezan-Roșu, Nicoleta Jemna, Irina Benedek, Julia Rednic, Irina Maria Vlad, Ana-Maria Buruiană, Ioana Mureșanu, Diana Chira, Livia Livinț Popa, Adina Dora Stan, Herbert Moessler, Ștefan Strilciuc

**Affiliations:** 1 Department of Neuroscience, Iuliu Hatieganu University of Medicine and Pharmacy, Victor Babes 8, 400347 Cluj-Napoca, Romania; dafinm@ssnn.ro (D.M.); olivia.rosu@brainscience.ro (O.V.-R.); irina.benedek@brainscience.ro (I.B.); irina.vlad001@gmail.com (I.M.V.); livia.popa@brainscience.ro (L.L.P.); adinadora@yahoo.com (A.D.S.); 2 RoNeuro Institute for Neurological Research and Diagnostic, Mircea Eliade 37, 400364 Cluj-Napoca, Romania; nicoleta.jemna@brainscience.ro (N.J.); juliarednic@gmail.com (J.R.); anamaria.buruiana@brainscience.ro (A.-M.B.); ioana.muresanu@brainscience.ro (I.M.); stefan.strilciuc@brainscience.ro (Ș.S.); 3 COMAMO Lifesciences GmbH, 4893 Zell am Moos, Austria; herbert.moessler@comamo.at; 4 Research Center for Functional Genomics, Biomedicine and Translational Medicine, Iuliu Hatieganu University of Medicine and Pharmacy, Gheorghe Marinescu 32-38, 400000 Cluj-Napoca, Romania

**Keywords:** supratentorial ischemic stroke, cognitive recovery, N-Pep-12, dietary supplementation, neuropsychological performance, post stroke cognitive impairment, neurorecovery, MoCA, vascular cognitive impairment

## Abstract

**Background**: Cognitive deficits following ischemic stroke significantly impair quality of life, highlighting the need for effective interventions. This study evaluates the efficacy and safety of extended N-Pep-12 dietary supplementation in enhancing cognitive recovery post-stroke. **Methods**: In this randomized, open-label, controlled study, 106 patients with supratentorial ischemic stroke were enrolled to receive either 90mg N-Pep-12 or no supplementation daily for 360 days and were followed-up for 360 days. Cognitive function and emotional well-being were assessed using established neuropsychological scales at baseline, 90 days, and 360 days post-stroke. Safety was monitored through adverse events and mortality rates. **Results**: Significant improvements were observed in the N-Pep-12 group compared to controls, particularly in the Montreal Cognitive Assessment scores at both 90 and 360 days, and in the Digit Symbol Coding scores at 360 days, suggesting enhanced cognitive recovery with extended N-Pep-12 supplementation. A linear regression for a composite outcome analysis at day 360 further confirmed the efficacy of N-Pep-12 in contributing to cognitive improvement. Safety profiles were favorable, with no significant adverse effects attributed to N-Pep-12. **Conclusions**: Extended dietary supplementation with N-Pep-12 appears to offer a safe and effective approach to support cognitive recovery in ischemic stroke survivors. These findings underscore the potential of the supplement as an add-on intervention for managing post-stroke cognitive impairments.

## 1. Introduction

Stroke is a serious medical condition, being the second cause of death worldwide. It occurs when the blood supply to a part of the brain is either reduced or completely interrupted. There are two main categories in which stroke is broadly classified: ischemic stroke, when a blockage in a blood vessel restricts blood flow to the brain and hemorrhagic stroke, when a blood vessel ruptures, leading to bleeding within the intracranial space [1] A supratentorial ischemic stroke refers specifically to an ischemic event that occurs in the brain region located above the tentorium cerebelli due to restriction of blood supply in that area [2].

Stroke survivors often experience cognitive deficits that span a broad spectrum, notably impacting their daily functioning, including living independently, driving, maintaining relationships and overall quality of life [3,4,5]. These deficits, which encompass a large range of cognitive impairments such as mild cognitive impairment (MCI) to severe dementia, including post stroke cognitive impairment (PSCI) are classified under the umbrella of vascular cognitive impairment (VCI). The patient’s individual cognitive deficits post stroke are heterogenous and the treatment options are scarce, with no pharmacological treatment approved for PSCI [6] or VCI [7,8]. With a prevalence of PSCI of up to 80% [9] and VCI being reported for a minimum of 20-40% of all diagnoses of dementia [10] this stark statistic underscores the pressing need for effective therapeutic interventions aimed at mitigating these cognitive challenges [4,6,8,11].

This study explored the potential of N-Pep-12 dietary supplementation in enhancing the recovery of individuals with cognitive impairments post-stroke.

N-Pep-12, a proprietary peptide-based nutritional supplement, has been previously shown to exert neuroprotective and cognitive-enhancing effects in both preclinical and clinical studies [12,13,14,15,16]. N-Pep-12 has been shown to exert dose-dependent neuroprotection against various types of lesions in neuronal cultures, suggesting its potential to prevent neuronal cell death associated with conditions like ischemic stroke and other neurological disorders [12]. Furthermore, the dietary supplement pointed towards an improvement in memory and other cognitive abilities among healthy older adults experiencing age-related memory loss. In this randomized, open label comparison, subjects treated with N-Pep-12 performed better on cognitive assessments than those receiving a placebo, suggesting that N-Pep-12 may be an effective supplement for ameliorating memory loss in this population [13].

The present study’s original pilot suggested a benefit of dietary supplementation up to 90 days with N-Pep-12 for the support of neurorecovery after supratentorial ischemic stroke mirrored in the observed improvements in neuropsychological outcome scales [14,15]. The same population showed that N-Pep-12 supplementation (90mg) might have a strengthening effect on post stroke neurorecovery as emphasized by its significant impact on quantitative electroencephalography (QEEG) parameters in patients after supratentorial ischemic stroke [16]. A similar study, conducted on healthy elderly subjects, investigated the effects of the supplementation with a one-time oral dose (180mg) on brain bioelectrical activity and cognitive performance. The study found that N-Pep-12 significantly increased relative alpha-activity power, an EEG rhythm typically associated with a relaxed and alert state. It also decreased slow delta-activity, which is often linked to deep sleep or pathological conditions [17]. These results indicate that N-Pep-12 might be beneficial for improving and maintaining brain function among healthy older adults [18].

These studies significantly contribute to the growing body of evidence supporting the cognitive benefits of N-Pep-12, particularly in promoting neurorecovery among older adults and patients recovering from ischemic stroke. While previous research is promising, the observed benefits often reflect a descriptive superiority over control groups rather than statistically conclusive outcomes. As such, a valid hypothesis is whether extending treatment beyond the original 90-day regimen would yield better results. The primary goal of this exploratory study was to evaluate the therapeutic efficacy and safety of a 360-day, once-daily regimen of 90 mg N-Pep-12 for post-stroke cognitive recovery, against a control group.

## 2. Materials and Methods

### 2.1. Study Procedures

This was a prospective, randomized, open-label, controlled study conducted at the “RoNeuro” Institute for Neurological Research and Diagnostic in Cluj-Napoca, Romania between 27 April 2020 and 31 October 2022. The study assessed the efficacy and safety of N-Pep-12 in neurorecovery post- supratentorial ischemic stroke over 360 days. Regular marketable 90 mg capsules of N-Pep-12 (Cebrium) were used in this study, administered once daily in the treatment group. The control group did not receive any study-specific medication or placebo during the trial.

The trial received approval from the Ethics Committee of the Iuliu Hatieganu University of Medicine and Pharmacy (approved 27 March 2020, +40 (0)264 597 256; contact@umfcluj.ro, ref: 115/16.03.2020) and adhered to ICH-GCP (International Council for Harmonisation—Good Clinical Practice) guidelines and the Declaration of Helsinki. The study was prospectively registered in the ISRCTN (International Standard Randomised Controlled Trial Number) registry [15].

All participants provided signed informed consent before participation. Individuals meeting the inclusion and exclusion criteria were assigned to the treatment group or control group, in a 2:1 ratio to motivate participation of patients. As a single blinded study, communication was forbidden between investigators performing assessments and the person who allocated the treatment.

### 2.2. Inclusion and Exclusion Criteria

Participants eligible for this study were adults between the ages of 18 and 80 who had experienced a supratentorial ischemic stroke located in the supratentorial region, with the onset occurring 30 to 120 days prior to screening. This stroke must have been radiologically confirmed via CT or MRI scans. Candidates were required not to have significant disabilities prior to their stroke, as evidenced by a pre-stroke Modified Rankin Score [19] of 0 or 1 and to have a Goodglass and Kaplan Communication Scale Score greater than 2 at screening [20]. Additionally, they must not have had another radiologically confirmed supratentorial stroke in the three months preceding the index stroke and needed to provide signed informed consent.

Exclusion criteria for the study included individuals with active, pre-existing major neurological diseases or psychiatric conditions such as major depression, bipolar disorder, schizophrenia, or dementia, as indicated by a short Informant Questionnaire on Cognitive Decline in the Elderly (IQCODE) score greater than 3 [21]. Candidates were also excluded if they had advanced liver, pulmonary, kidney, or cardiac disease; a terminal medical diagnosis indicating a life expectancy of less than one year; a major dependency on drugs, including alcohol, as judged by the investigator; any injury to the writing hand that could influence cognitive or other outcome measures; or if they were pregnant or lactating women. There were no restrictions on concomitant treatments or therapies for the study participants and all such treatments or therapies were recorded in the patient’s CRF.

### 2.3. Study Assessments

Primary outcomes focusing on cognitive function and emotional well-being in stroke patients were measured using five established neuropsychological scales (MoCA, Montreal Cognitive Assessment; HADS, Hospital Anxiety and Depression subscales; CTT, Color Trails Test; DSF, Digit Span Forward; DSB, Digit Span Backward; PSI, Processing Speed Index (subscales DSC, Digit Symbol Coding; SS, Symbol Search correct; SSI, Symbol Search incorrect) from the Wechsler Adult Intelligence Scale, 3rd edition), measured at baseline, 90 days, and 360 days after study inclusion [22,23,24,25,26].

Secondary outcomes addressed safety through adverse events (AEs), serious adverse events (SAEs) and mortality throughout the study, ensuring comprehensive monitoring of patient wellbeing and the intervention’s impact. An adverse event (AE) was considered any untoward medical occurrence encountered in a patient who received the supplement product and is not necessarily caused by this treatment. An adverse drug reaction was considered as serious if it resulted in patient’s death, was life threatening, it required additional or prolonged inpatient hospitalization or it led to a lasting or serious disability or other important medical issue that requires urgent medical or surgical intervention

### 2.4. Study Visits

During the first study visit (screening/baseline at 30–120 days post-stroke), participants were briefed about the study’s purpose and procedures. Following their informed consent, eligibility was confirmed via assessment against the study’s inclusion and exclusion criteria. Eligible candidates were then randomized and assigned a unique code. Baseline data collection entailed recording medical history, vital signs, demographic details, and any concurrent medications, all of which were meticulously documented in the paper-based case report form (CRF). Baseline evaluations for both primary and secondary outcome measures were conducted, with findings duly recorded in the CRF. Participants commenced their treatment regimen with the first dose of the study drug, continuing for the initial 90 days of the study period. At Day 90, participants underwent a repeat measurement of vital signs and any alterations in their medication regimens since the last visit were updated in the CRF. The session also included a thorough review of any adverse events reported by participants or observed by researchers, ensuring accurate and comprehensive documentation in the CRF. Adherence to the study medication was verified, with findings noted in the CRF. The treatment course was extended for an additional 270 days. At Day 360, a comprehensive health review was repeated, including updating vital signs and any changes in medication use. The assessment and documentation process for any reported or observed adverse events were conducted with the same rigor as in previous visits, ensuring thorough record-keeping in the CRF. Medication compliance was once again verified. The final round of evaluations for the study’s primary and secondary efficacy metrics was carried out identically to day 90.

### 2.5. Quality Assurance and Data Analysis

Investigators allowed monitoring, audits, and regulatory inspections, providing access to source data that supported CRF data. Source data were defined as all information in the original records and certified copies of original records of clinical findings, observations or other activities in a clinical study necessary for the reconstruction and evaluation of the study. Quality control processes were implemented at every data handling stage to ensure reliability, with regular site visits by the study coordinator to ensure protocol adherence. Quality assurance measures were in place to comply with good clinical practice and regulatory standards.

The study’s data were analyzed, with the statistical report having been compiled after all data were entered and validated in a spreadsheet database. The analysis population was defined as all subjects who did not show significant protocol deviations or missing data. The safety population included all participants who had a minimum of one treatment dose and one subsequent visit with study investigators. Variables were computed for change across baseline, day 90 and day 360 evaluations by subtracting the scores obtained at the baseline from the corresponding efficacy visit.

The statistical analysis for the study was conducted using SAS University Edition. This involved descriptive statistics to summarize the data, Mann–Whitney and *t*-tests for group comparisons, and linear regression to assess the effect of N-Pep-12 on composite neuropsychological outcomes. In constructing composite dependent variables, we first standardized the scores from the five primary outcome scales (MoCA, HADS, CTT, DS, and PSI) to z-scores, adjusting for differences in their scoring ranges and units. These standardized scores were then averaged for each participant to create a single composite score. For subscales, a weighted average was calculated. This composite score served as the dependent variable during further analyses. Standard assumptions (e.g., normality) were tested and a two-sided alpha level of 0.05 was used to determine statistical significance.

## 3. Results

In total, 106 patients were enrolled in both groups, with dropouts due to lost-to-follow-up where patients did not attend the second visit (n = 7) or had missing data (n = 8). Of these, 91 patients who received N-Pep-12 (n = 58) or were assigned as controls (n = 33) were included in the analysis set.

In total, 18 AEs were reported throughout the study. The N-Pep-12 group accounted for 14 of these events, while the control group reported 4 adverse events. The types of AEs observed in the N-Pep-12 group included vertigo, dizziness, confusion, loss of appetite, and cases of COVID-19, among others. All AEs were monitored closely and appropriate medical care was provided when necessary. In cases such as dizziness and confusion, patients were advised to rest and were followed up to ensure symptoms resolved. It is important to note that, as per the study protocol, any medical event occurring after informed consent is classified as an AE, regardless of its relation to the study medication. The cases of COVID-19 were deemed unrelated to the study treatment and were recorded as general AEs, not adverse drug reactions. Fisher’s exact test revealed no statistically significant difference in the incidence of AEs between the two groups (*p* = 1.0). This suggests that the increased number of AEs in the N-Pep-12 group may not be of clinical significance and could be due to chance. Regarding SAEs, three SAEs were reported in the N-Pep-12 group: memory impairment, myocardial infarction and ischemic stroke. In contrast, the control group reported two SAEs: a right olecranon fracture and atrial fibrillation. To determine the significance of these differences, Fisher’s exact test was performed, yielding a *p*-value of 0.533 and an odds ratio of 0.273. These results indicate that the difference in SAE incidence between the N-Pep-12 and control groups is not statistically significant, further supporting the conclusion that N-Pep-12 does not pose a higher risk of serious adverse events compared to the control treatment.

**Figure 1:**
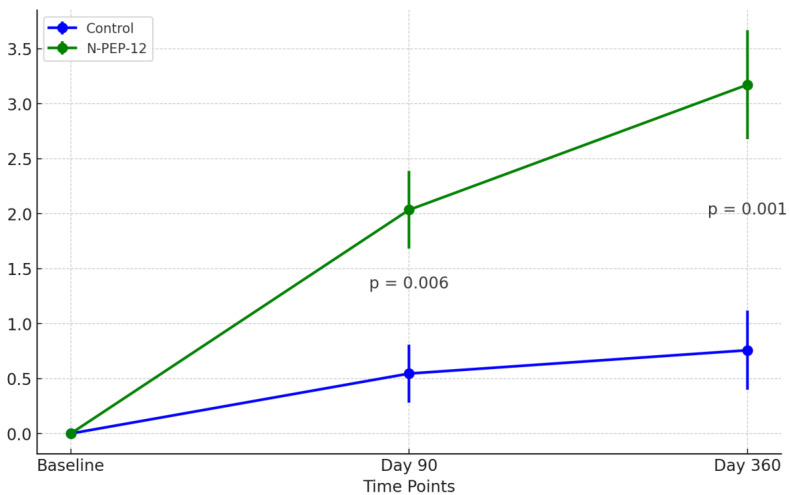
Comparison of MoCA evolution from baseline (differences) across study subjects and groups; *p*-values represent nonparametric group differences; vertical bars are standard errors for the mean (point estimates).

Minimum, maximum, mean, median and standard deviation values of neuropsychological outcome scales across study groups at baseline are available in Table 1. Additionally, Figure 1 illustrates the comparison of MoCA evolution from baseline across study groups.

The results of the clinical trial were analyzed for several key outcome variables. The tests for normality, *t*-tests, and nonparametric Wilcoxon tests were performed for each variable at 90 days and 360 days. All analyses were reported based on non-parametric test values due to non-normal distributions of the data. Significant differences between groups were noted for multiple outcome scales and timepoints in favor of the intervention, suggesting areas of notable change or effect.

The MoCA scores, a measure of overall cognitive function, increased significantly in the NPEP group compared to the no supplementation group at 90 days (mean difference = 1.49, 95% CI [0.53, 2.45], *p* = 0.003) and 360 days (mean difference = 2.41, 95% CI [1.21, 3.62], *p* < 0.001). The DSC scores increased significantly in the NPEP group compared to the placebo group at 360 days (mean difference = 4.27, 95% CI [0.75, 7.79], *p* = 0.018), but there was no significant difference between groups at 90 days (mean difference = −2.33, 95% CI [−6.26, 1.61], *p* = 0.243). The SS scores, also increased significantly in the NPEP group compared to the control group at 360 days (mean difference = 2.97, 95% CI [1.40, 4.55], *p* = 0.0003), but there was no significant difference between groups at 90 days (mean difference = 0.59, 95% CI [−1.14, 2.32], *p* = 0.502). There was no significant difference between groups in Digit Span (DSF; 90 days: mean difference = 0.12, 95% CI [−0.73, 0.96], *p* = 0.783; 360 days: mean difference = 0.62, 95% CI [−0.26, 1.50], *p* = 0.167); DSB; 90 days: mean difference = −0.28, 95% CI [−1.04, 0.46], *p* = 0.454; 360 days: mean difference = 0.62, 95% CI [−0.11, 1.34], *p* = 0.093), or Processing Speed Index errors (SSI; 90 days: mean difference = −0.20, 95% CI [−1.07, 0.66], *p* = 0.640; 360 days: mean difference = −0.20, 95% CI [−1.01, 0.60], *p* = 0.615) at 90 or 360 days. A complete account of the statistical tests is available in the Supplementary Materials. A full account of group difference tests is also available in the Supplementary Materials.

A linear regression was conducted to assess the relationship between supplement use (NPEP vs. control) and composite cognitive outcomes (z-scores) at day 360 revealed a significant effect of the supplement use (F(1, 89) = 12.71, *p* = 0.0006). Participants in the NPEP group (estimate = 3.27, *p* = 0.0006) had significantly higher composite cognition scores compared to controls at the study endpoint (day 360). The model accounted for 12.5% of the variance in outcome scores. A full description of the regression procedures is available as Supplementary Materials.

## 4. Discussion

Post-stroke cognitive dysfunction has a high prevalence, impacting patients’ quality of life. The diagnosis encompasses a comprehensive evaluation (neurocognitive assessment and paraclinical tests) assessing the severity of the existing deficits, causes and treatment planning. Considering the lack of approved medication, with acetylcholinesterase inhibitors having shown modest cognitive improvements in conducted studies, an important aspect regarding the management of post stroke cognitive dysfunction is represented by cognitive rehabilitation, control of modifiable risk factors and comorbidities, support for patients and caregivers and pharmacological interventions. Nevertheless, considering the complexity of the disorder, further studies are required [6,27,28,29,30,31,32].

This study aimed to investigate the long-term effects of N-Pep-12 dietary supplementation on neurorecovery and cognitive performance among individuals who have suffered from supratentorial ischemic stroke. Considering the results obtained following the 90-days administration of 90 mg of N-Pep-12 daily [14], the current study sought to provide a more comprehensive understanding of the potential benefits of N-Pep-12 in promoting cognitive recovery post-stroke by extending the supplementation period beyond 90 days to a total of 360 days.

Our findings revealed a nuanced landscape of cognitive recovery facilitated by N-Pep-12 supplementation. At both the 90-day and 360-day marks, significant improvements were noted in total MoCA scores and in the DSC and CTT at 360 days, suggesting that extended supplementation may contribute to sustained cognitive recovery. The significant differences in these cognitive tests between the treatment groups suggest that the N-Pep treatment leads to better cognitive outcomes in some areas compared to no supplementation, aligning with the neuroprotective and cognitive-enhancing properties of N-Pep-12 observed in previous studies, hence emphasizing its potential in supporting cognitive function and recovery after ischemic stroke [14,16].

The statistical analyses, incorporating both *t*-tests and non-parametric tests due to the violation of normality in some variables, underscored the importance of a tailored approach to cognitive recovery. While some scales, such as DSB, DSF, and HADS, only showed descriptive differences between the treatment and control groups in the studied population, the notable improvements in MoCA and CT2 scales indicate that N-Pep-12 supplementation may have specific areas of impact within the broader spectrum of cognitive functions affected by supratentorial stroke. Moreover, the study’s linear regression analysis, predicting the composite dependent variable at Day 360, further illustrated the potential efficacy of N-Pep-12 in enhancing cognitive outcomes post-stroke. The model’s significant prediction of the dependent variable suggests that N-Pep-12 supplementation plays a notable role in enhancing cognitive recovery. The safety profile of N-Pep-12, assessed through the monitoring of AEs, SAEs and mortality, remained favorable throughout the study. The few instances of SAEs and the overall compliance with the supplementation regimen reinforce the feasibility of extending N-Pep-12 supplementation as a safe and potentially effective approach to post-stroke cognitive recovery.

This study has several limitations. The small sample size may have reduced our ability to detect subtle yet clinically important differences between the groups. Additionally, conducting the study at a single center may have limited how generalizable our findings are. Variability in the time from stroke onset to baseline assessment, along with the absence of patients with severe post-stroke disabilities, may have contributed to sample heterogeneity. Furthermore, unmeasured factors like social support and access to rehabilitation were not systematically documented and may have affected cognitive recovery. Nevertheless, some of these limitations adhere to the exploratory nature of the study as well as the fact that the study was conducted during the COVID-19 pandemic. The suggested benefits of N-Pep-12 administration observed in patients with cognitive impairment post stroke in this study should stimulate interest in further large-scale studies to confirm these findings.

## 5. Conclusions

In conclusion, the N-Pep-12 Extension Study offers valuable insights into the long-term benefits of N-Pep-12 dietary supplementation in the context of supratentorial stroke recovery. While the differential effects across various cognitive scales highlight the complexity of post-stroke cognitive impairment, the significant improvements in key areas suggest a promising role for N-Pep-12 in enhancing cognitive recovery and overall quality of life for stroke survivors.

Future multi-centered studies with larger sample sizes that are double blinded, address all stroke-severities, and focus on delineating the specific cognitive domains most responsive to N-Pep-12 supplementation will be crucial for confirming these results, as well as optimizing post-stroke rehabilitation strategies and maximizing recovery outcomes.

## Figures and Tables

**Table 1:** Descriptive values of neuropsychological outcome scales at baseline (mean ± standard deviation).

Variable	Intervention (n = 58)	Control (n = 33)
MoCA	22.2 ± 5.0	25.0 ± 3.8
HADS-A	7.0 ± 4.1	6.6 ± 3.5
HADS-D	5.7 ± 3.9	5.8 ± 4.5
CTT	184.9 ± 109.3	146.7 ± 72.8
DSF	6.9 ± 2.4	7.6 ± 2.6
DSB	4.6 ± 2.1	5.3 ± 2.1
DSC	31.5 ± 18.0	37.8 ± 16.7
SS	15.2 ± 7.4	17.2 ± 8.0
SS incorrect	2.6 ± 2.2	2.1 ± 2.0

Abbreviations: MoCA—Montreal Cognitive Assessment; HADS-A—Hospital Anxiety and Depression—Anxiety Subscale; HADS-D–Hospital Anxiety and Depression–Depression Subscale; CTT—Color Trails Test; DSF—Digit Span Forward; DSB—Digit Span Backward; DSC—Digit Symbol Coding; SS—Symbol Search.

## Data Availability

Dataset is available upon reasonable request.

## Supplementary Materials

The supplementary material can be downloaded at: https://www.mdpi.com/article/10.3390/brainsci14100986/s1.
