# Supplementary material for: Neuropsychological Performance after Extended N-Pep-12 Dietary Supplementation in Supratentorial Ischemic Stroke
Source: Brain Sci. 2024 Sep 28;14(10):986. doi: 10.3390/brainsci14100986 (PMC11506754; doi:10.3390/brainsci14100986)
Supplement: Supplementary file 1 [file brainsci-14-00986-s001.zip › brainsci-3217117-supplementary.pdf]

**

Medicatie=intervention - N_Pep_12
fara medicatie = control - placebo

Variable: MoCA90 (MoCA90)
MEDICATIE = NPEP

Tests for Normality				
Test	Statistic		p Value	
Shapiro-Wilk	W	0.970134	Pr < W	0.1626
Kolmogorov-Smirnov	D	0.108553	Pr > D	0.0877
Cramer-von Mises	W-Sq	0.10407	Pr > W-Sq	0.0978
Anderson-Darling	A-Sq	0.647153	Pr > A-Sq	0.0897

Variable: MoCA90 (MoCA90)
MEDICATIE = fara medicatie

Tests for Normality				
Test	Statistic		p Value	
Shapiro-Wilk	W	0.927726	Pr < W	0.0302
Kolmogorov-Smirnov	D	0.231444	Pr > D	<0.0100
Cramer-von Mises	W-Sq	0.216529	Pr > W-Sq	<0.0050
Anderson-Darling	A-Sq	1.052887	Pr > A-Sq	0.0082

Variable: MoCA90 (MoCA90)

MEDICATIE	Method	N	Mean	Std Dev	Std Err	Minimum	Maximum
NPEP		58	2.0345	2.6948	0.3538	-4.0000	9.0000
fara medicatie		33	0.5455	1.8890	0.3288	-5.0000	4.0000
Diff (1-2)	Pooled		1.4890	2.4359	0.5312		
Diff (1-2)	Satterthwaite		1.4890		0.4830		

MEDICATIE	Method	Mean	95% CL Mean		Std Dev	95% CL Std Dev	
NPEP		2.0345	1.3259	2.7430	2.6948	2.2782	3.2993
fara medicatie		0.5455	-0.1243	1.2153	1.8890	1.5191	2.4985
Diff (1-2)	Pooled	1.4890	0.4336	2.5444	2.4359	2.1247	2.8549
Diff (1-2)	Satterthwaite	1.4890	0.5286	2.4494			

Method	Variances	DF	t Value	Pr > |t|
Pooled	Equal	89	2.80	0.0062
Satterthwaite	Unequal	85.018	3.08	0.0028
Cochran	Unequal	.	3.08	0.0036

Equality of Variances				
Method	Num DF	Den DF	F Value	Pr > F
Folded F	57	32	2.04	0.0323

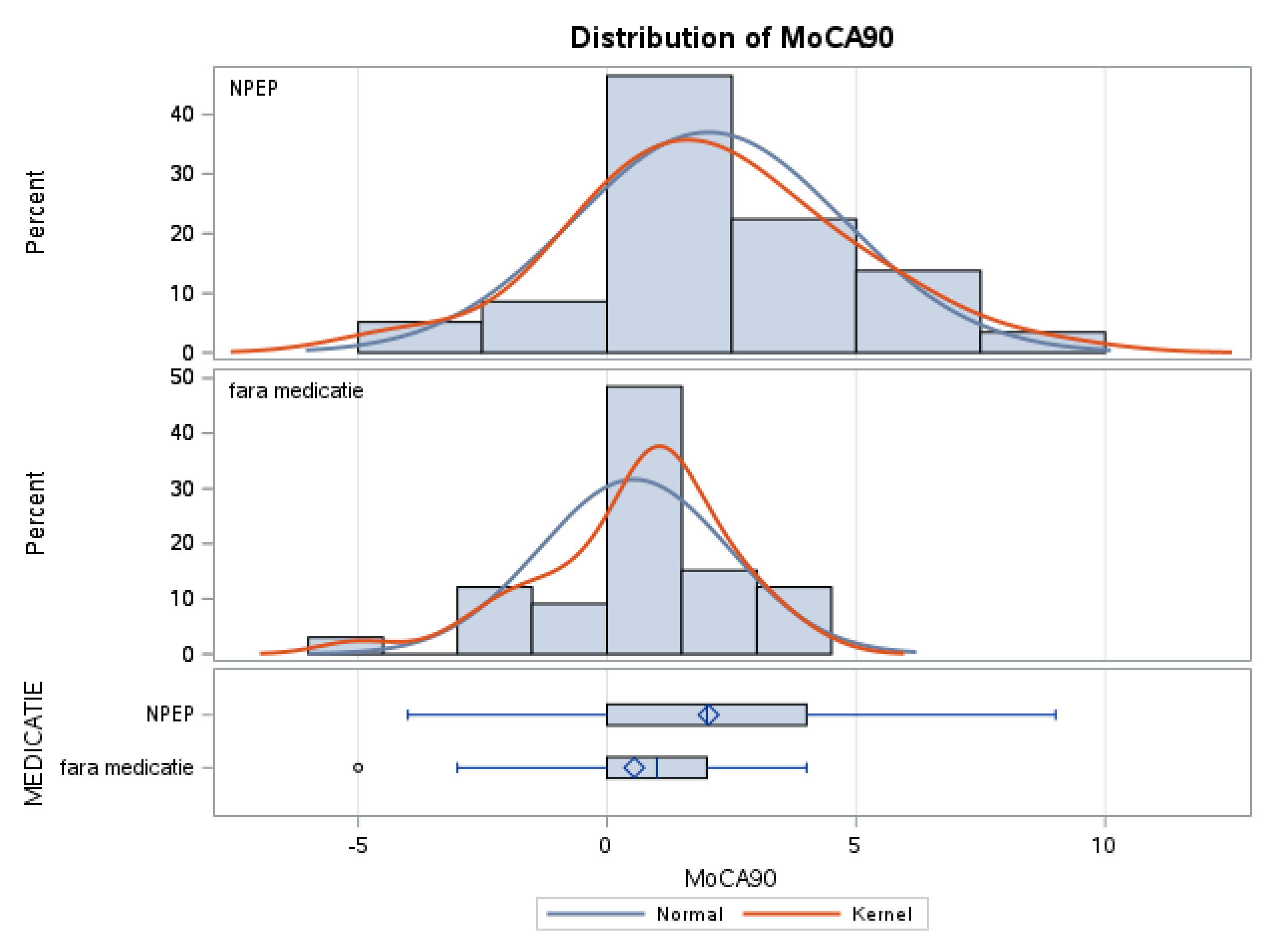
Distribution of MoCA90
NPEP
fara medicatie
Percent
40
30
20
10
0
50
MEDICATIE
NPEP
fara medicatie
-5
0
5
10
MoCA90
Normal
Kernel

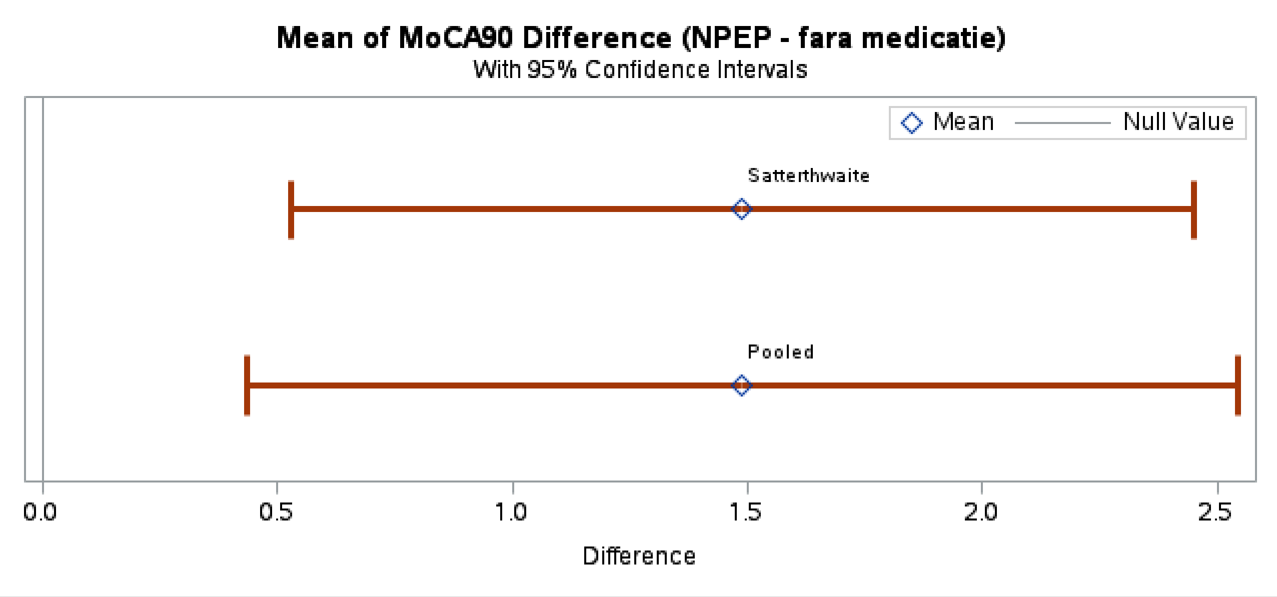
Mean of MoCA90 Difference (NPEP - fara medicatie)
With 95% Confidence Intervals
Mean
Null Value
Satterthwaite
Pooled
0.0
0.5
1.0
1.5
2.0
2.5
Difference

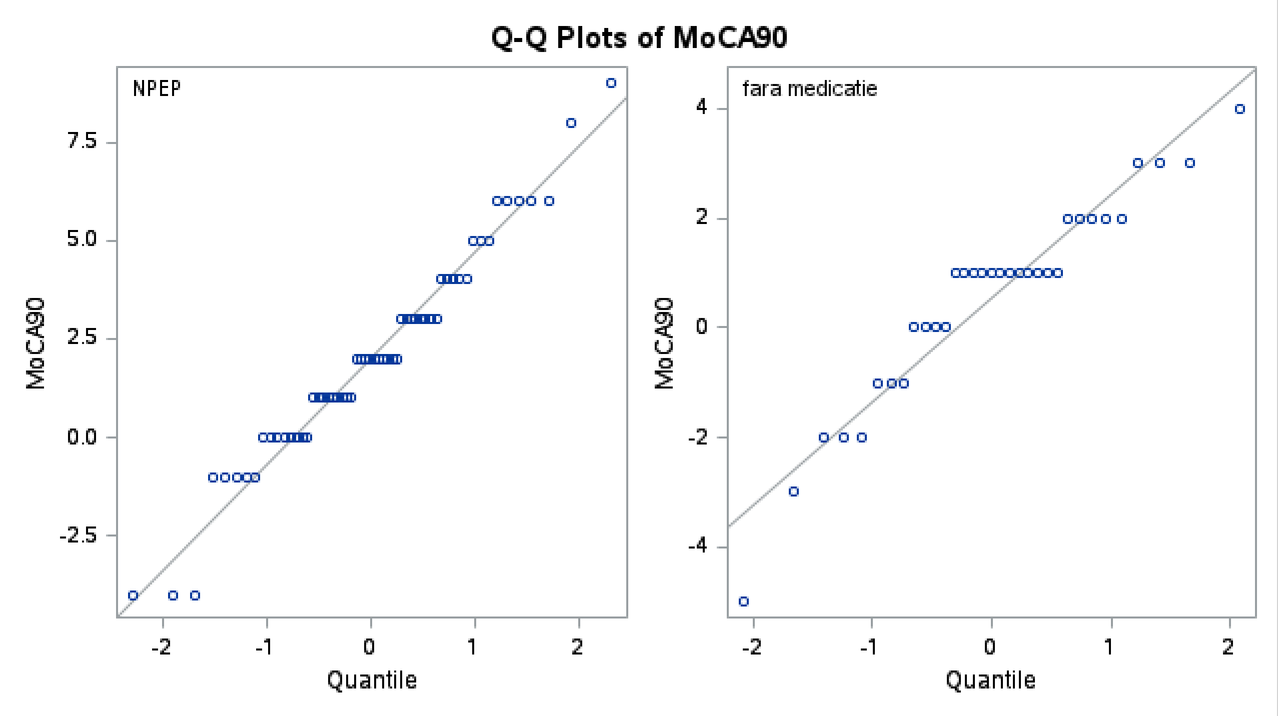

Wilcoxon Scores (Rank Sums) for Variable MoCA90 Classified by Variable MEDICATIE					
MEDICATIE	N	Sum of Scores	Expected Under H0	Std Dev Under H0	Mean Score
fara medicatie	33	1196.0	1518.0	119.799186	36.242424
NPEP	58	2990.0	2668.0	119.799186	51.551724
Average scores were used for ties.					

Wilcoxon Two-Sample Test					
				t Approximation	
Statistic	Z	Pr < Z	Pr > |Z|	Pr < Z	Pr > |Z|
1196.000	-2.6837	0.0036	0.0073	0.0043	0.0087
Z includes a continuity correction of 0.5.					

Kruskal-Wallis Test		
Chi-Square	DF	Pr > ChiSq
7.2244	1	0.0072

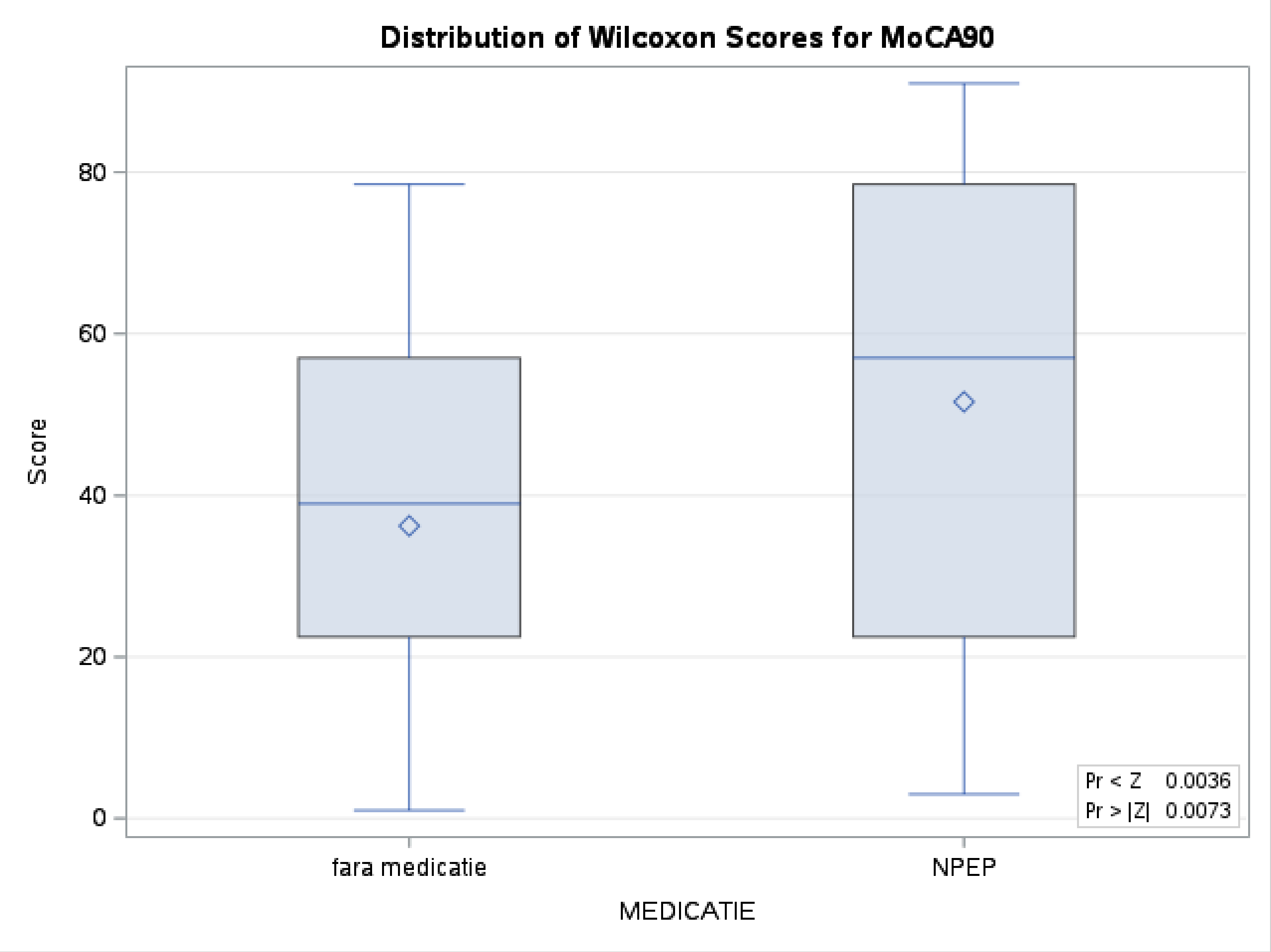

Variable: MoCA360 (MoCA360)
MEDICATIE = NPEP

Tests for Normality				
**Test**	**Statistic**		**p Value**	
**Shapiro-Wilk**	**W**	0.911662	**Pr < W**	0.0005
**Kolmogorov-Smirnov**	**D**	0.156276	**Pr > D**	<0.0100
**Cramer-von Mises**	**W-Sq**	0.274435	**Pr > W-Sq**	<0.0050
**Anderson-Darling**	**A-Sq**	1.69987	**Pr > A-Sq**	<0.0050

Variable: MoCA360 (MoCA360)
MEDICATIE = fara medicatie

Tests for Normality				
**Test**	**Statistic**		**p Value**	
**Shapiro-Wilk**	**W**	0.953488	**Pr < W**	0.1681
**Kolmogorov-Smirnov**	**D**	0.156964	**Pr > D**	0.0382
**Cramer-von Mises**	**W-Sq**	0.126843	**Pr > W-Sq**	0.0469
**Anderson-Darling**	**A-Sq**	0.718141	**Pr > A-Sq**	0.0566

Variable: MoCA360 (MoCA360)

MEDICATIE	Method	N	Mean	Std Dev	Std Err	Minimum	Maximum
**NPEP**		58	3.1724	3.7796	0.4963	-2.0000	13.0000

MEDICATIE	Method	N	Mean	Std Dev	Std Err	Minimum	Maximum
fara medicatie		33	0.7576	2.0005	0.3482	-3.0000	5.0000
Diff (1-2)	Pooled		2.4148	3.2539	0.7095		
Diff (1-2)	Satterthwaite		2.4148		0.6063		

MEDICATIE	Method	Mean	95% CL Mean		Std Dev	95% CL Std Dev	
NPEP		3.1724	2.1786	4.1662	3.7796	3.1953	4.6274
fara medicatie		0.7576	0.0482	1.4669	2.0005	1.6088	2.6460
Diff (1-2)	Pooled	2.4148	1.0051	3.8246	3.2539	2.8381	3.8136
Diff (1-2)	Satterthwaite	2.4148	1.2101	3.6196			

Method	Variances	DF	t Value	Pr > |t|
Pooled	Equal	89	3.40	0.0010
Satterthwaite	Unequal	88.662	3.98	0.0001
Cochran	Unequal	.	3.98	0.0002

Equality of Variances				
Method	Num DF	Den DF	F Value	Pr > F
Folded F	57	32	3.57	0.0002

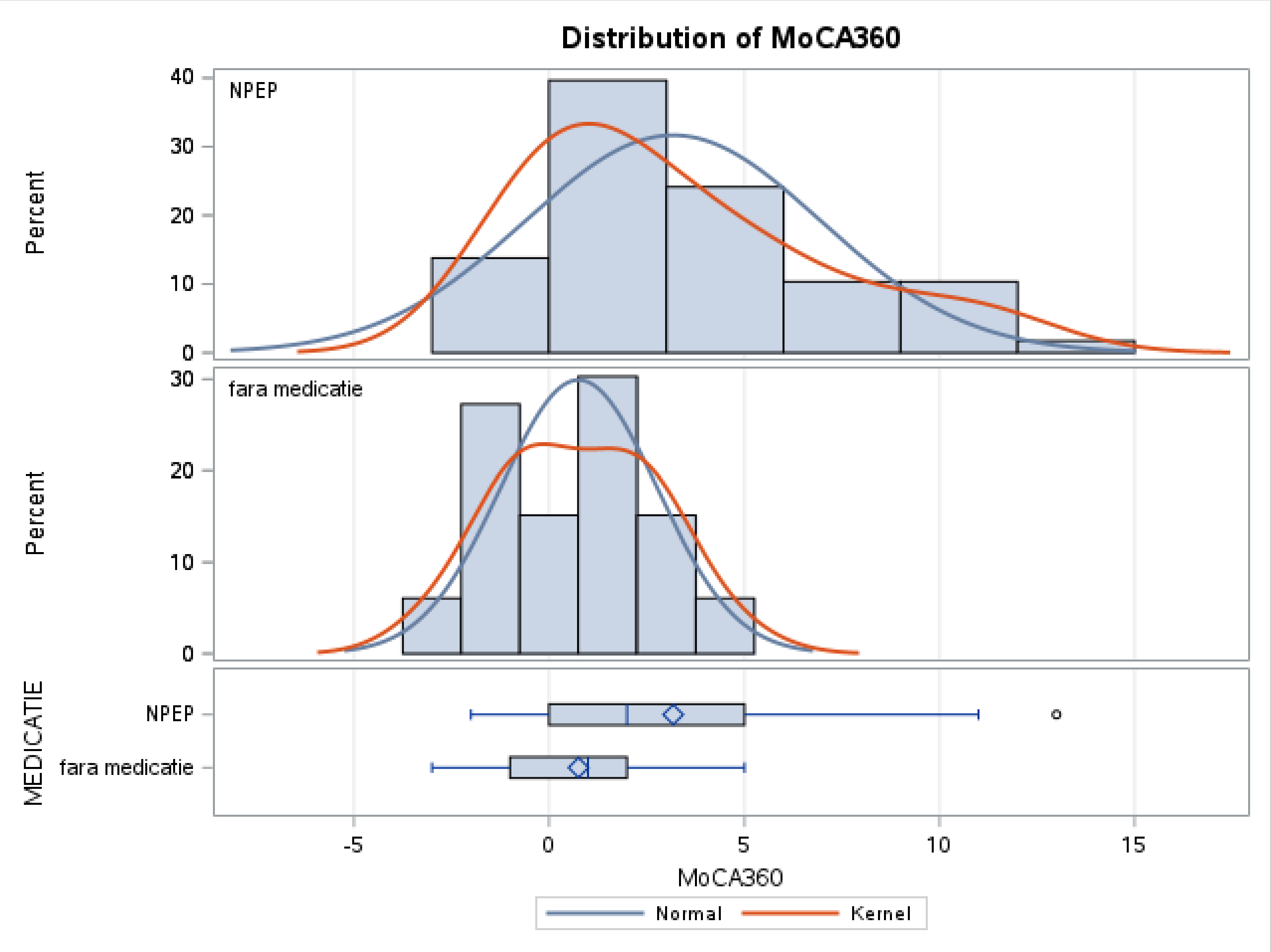

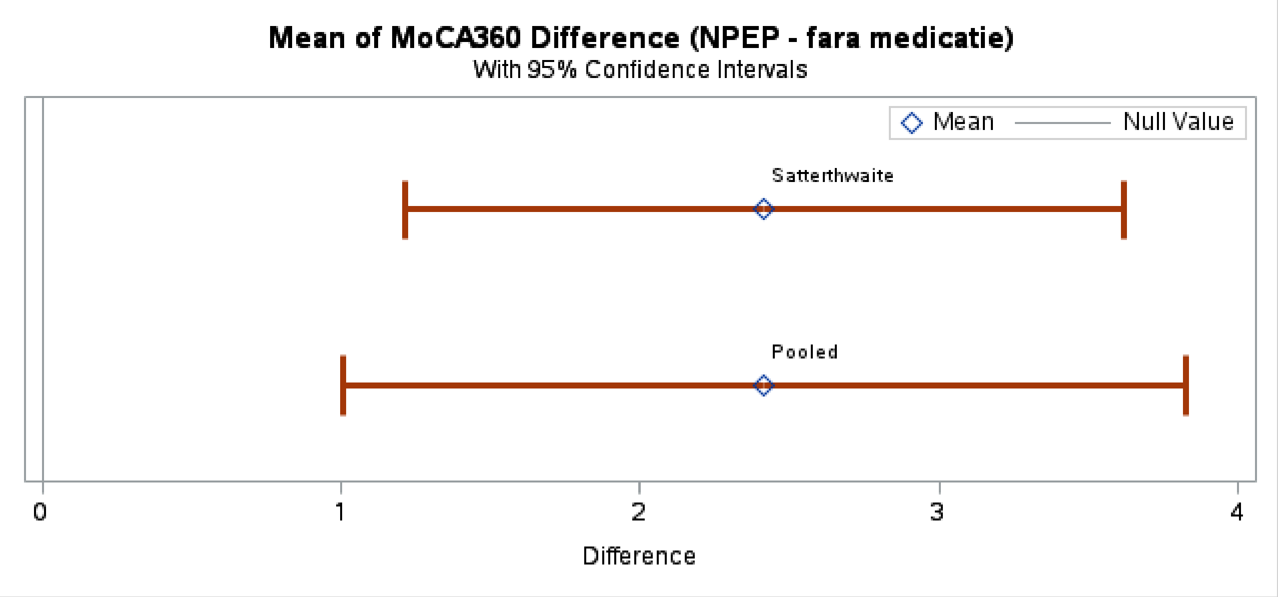

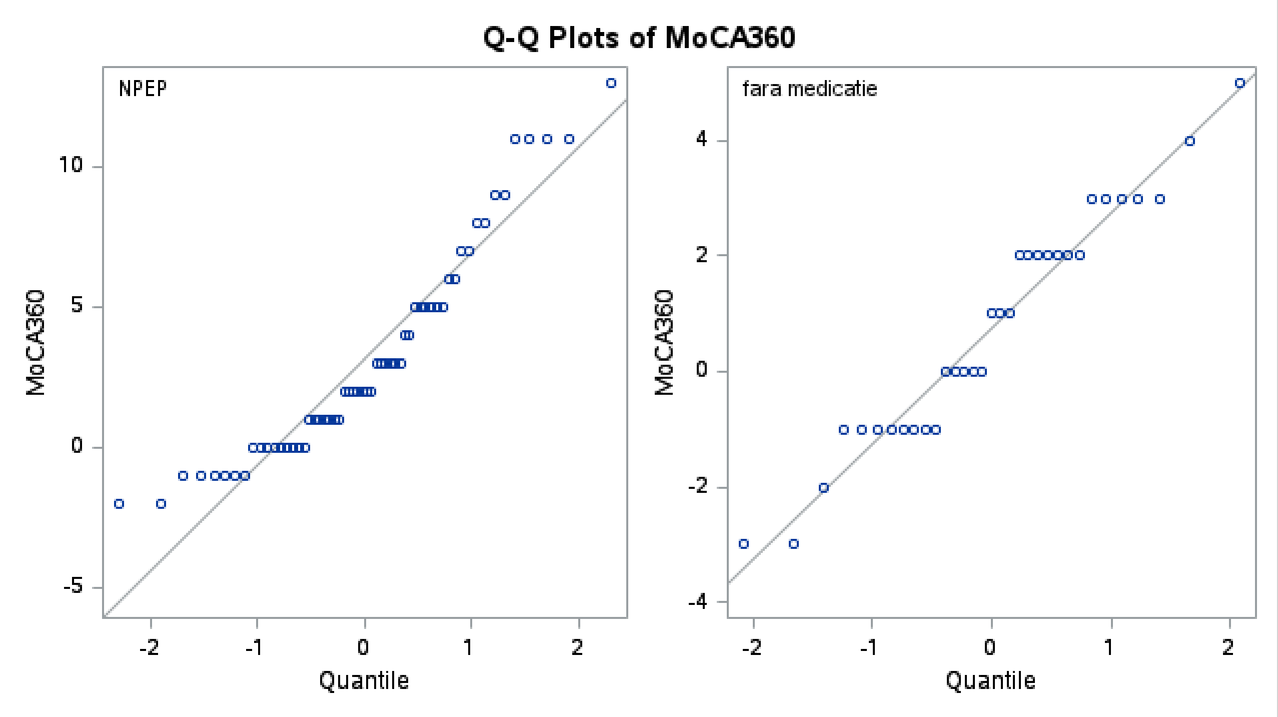

Wilcoxon Scores (Rank Sums) for Variable MoCA360 Classified by Variable MEDICATIE					
MEDICATIE	N	Sum of Scores	Expected Under H0	Std Dev Under H0	Mean Score
fara medicatie	33	1168.50	1518.0	120.250911	35.409091
NPEP	58	3017.50	2668.0	120.250911	52.025862
Average scores were used for ties.					

Wilcoxon Two-Sample Test					
				t Approximation	
Statistic	Z	Pr < Z	Pr > |Z|	Pr < Z	Pr > |Z|
1168.500	-2.9023	0.0019	0.0037	0.0023	0.0047
Z includes a continuity correction of 0.5.					

Kruskal-Wallis Test		
Chi-Square	DF	Pr > ChiSq

Kruskal-Wallis Test		
Chi-Square	DF	Pr > ChiSq
8.4473	1	0.0037

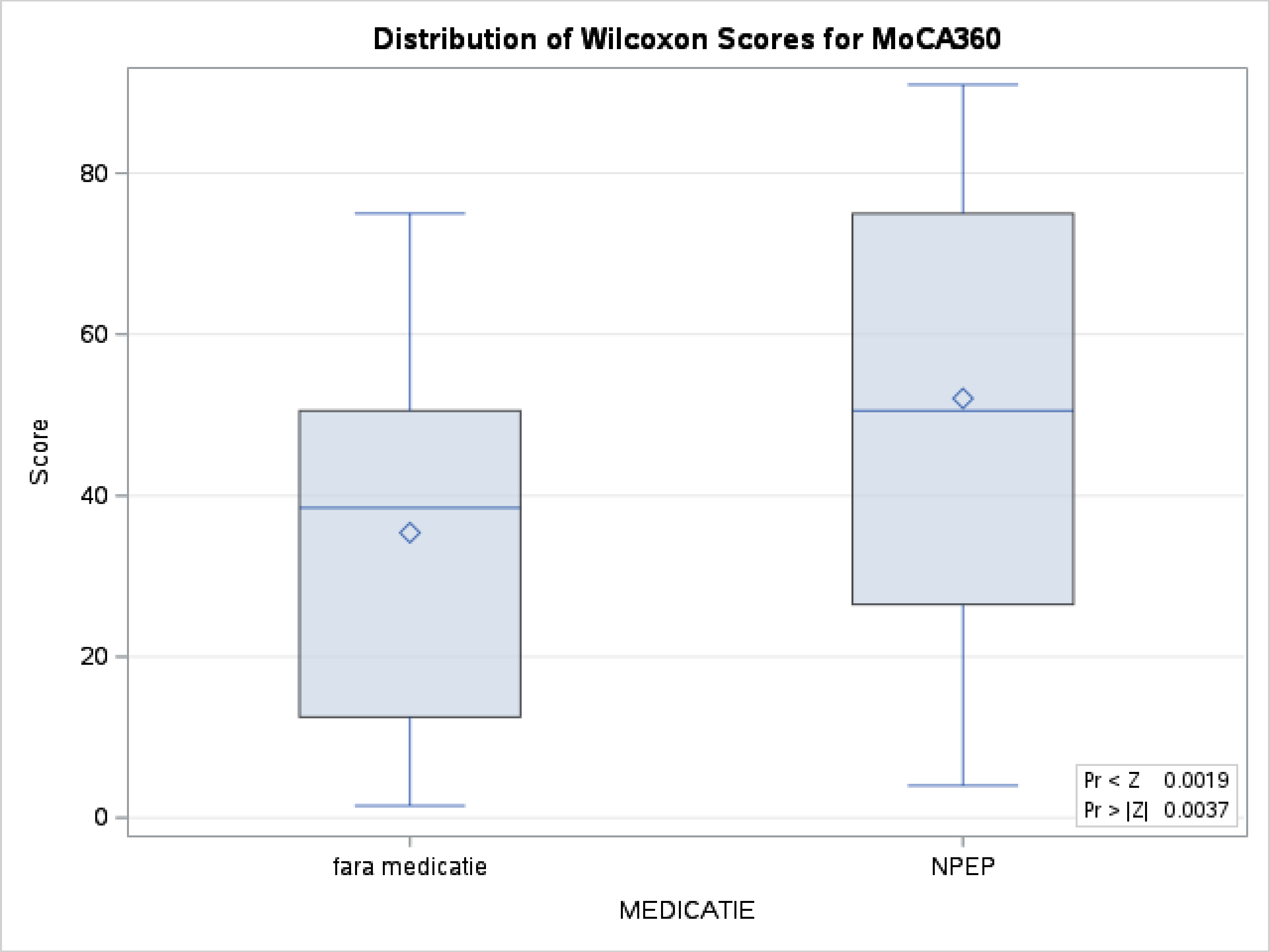

Variable: DSF90 (DSF90)
MEDICATIE = NPEP

Tests for Normality				
Test	Statistic		p Value	
Shapiro-Wilk	W	0.899022	Pr < W	0.0002
Kolmogorov-Smirnov	D	0.220336	Pr > D	<0.0100
Cramer-von Mises	W-Sq	0.42324	Pr > W-Sq	<0.0050
Anderson-Darling	A-Sq	2.210813	Pr > A-Sq	<0.0050

Variable: DSF90 (DSF90)
MEDICATIE = fara medicatie

Tests for Normality				
Test	Statistic		p Value	
Shapiro-Wilk	W	0.882299	Pr < W	0.0019
Kolmogorov-Smirnov	D	0.286133	Pr > D	<0.0100
Cramer-von Mises	W-Sq	0.385451	Pr > W-Sq	<0.0050
Anderson-Darling	A-Sq	1.884476	Pr > A-Sq	<0.0050

Variable: DSF90 (DSF90)

MEDICATIE	Method	N	Mean	Std Dev	Std Err	Minimum	Maximum
NPEP		58	-0.1552	2.0157	0.2647	-7.0000	4.0000
fara medicatie		33	-0.2727	1.8418	0.3206	-5.0000	3.0000
Diff (1-2)	Pooled		0.1176	1.9550	0.4263		
Diff (1-2)	Satterthwaite		0.1176		0.4157		

MEDICATIE	Method	Mean	95% CL Mean		Std Dev	95% CL Std Dev	
NPEP		-0.1552	-0.6852	0.3748	2.0157	1.7041	2.4679
fara medicatie		-0.2727	-0.9258	0.3803	1.8418	1.4811	2.4361
Diff (1-2)	Pooled	0.1176	-0.7294	0.9646	1.9550	1.7051	2.2912
Diff (1-2)	Satterthwaite	0.1176	-0.7113	0.9464			

Method	Variances	DF	t Value	Pr > |t|
Pooled	Equal	89	0.28	0.7834
Satterthwaite	Unequal	71.768	0.28	0.7782
Cochran	Unequal	.	0.28	0.7789

Equality of Variances				
Method	Num DF	Den DF	F Value	Pr > F
Folded F	57	32	1.20	0.5891

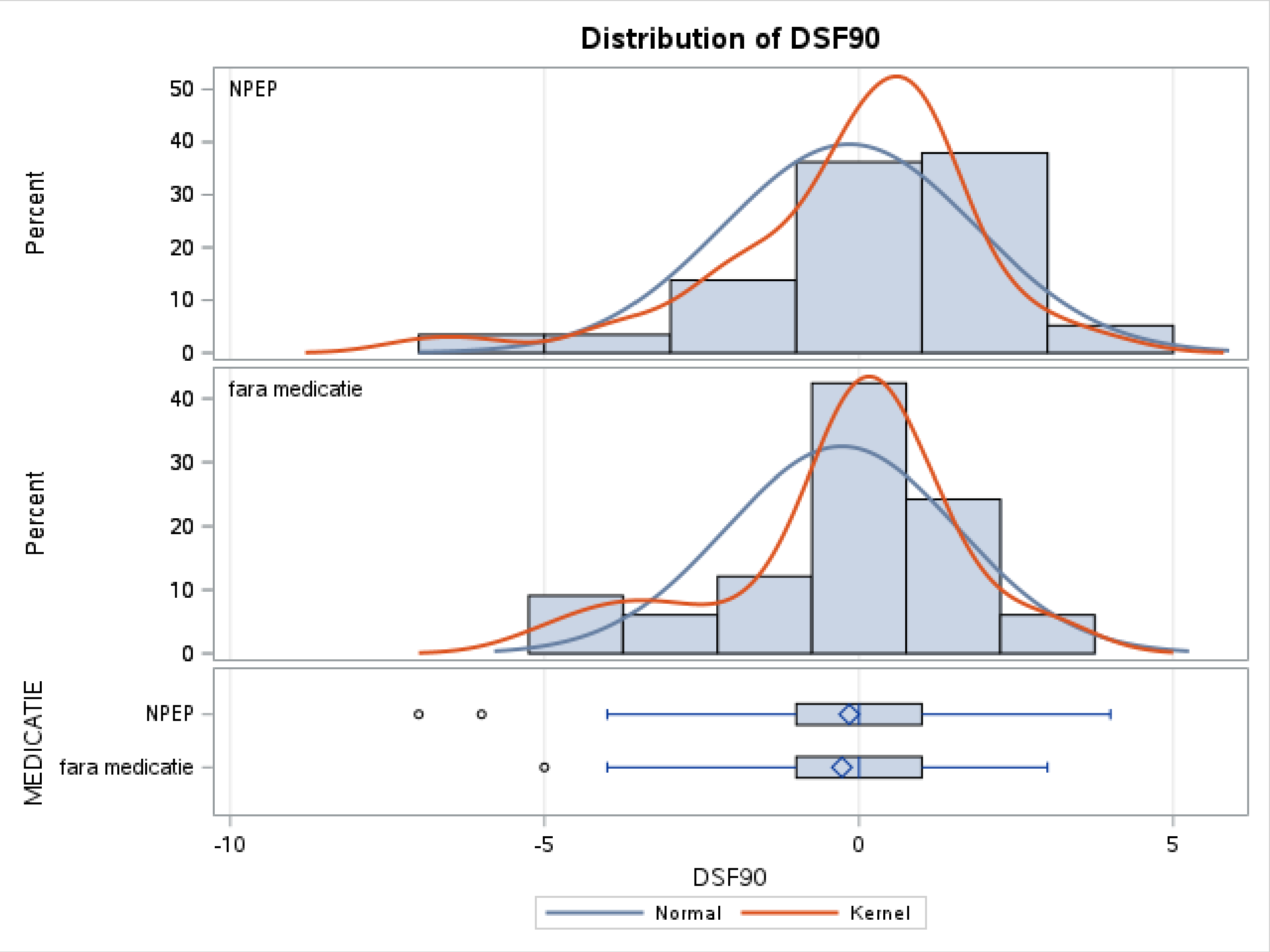

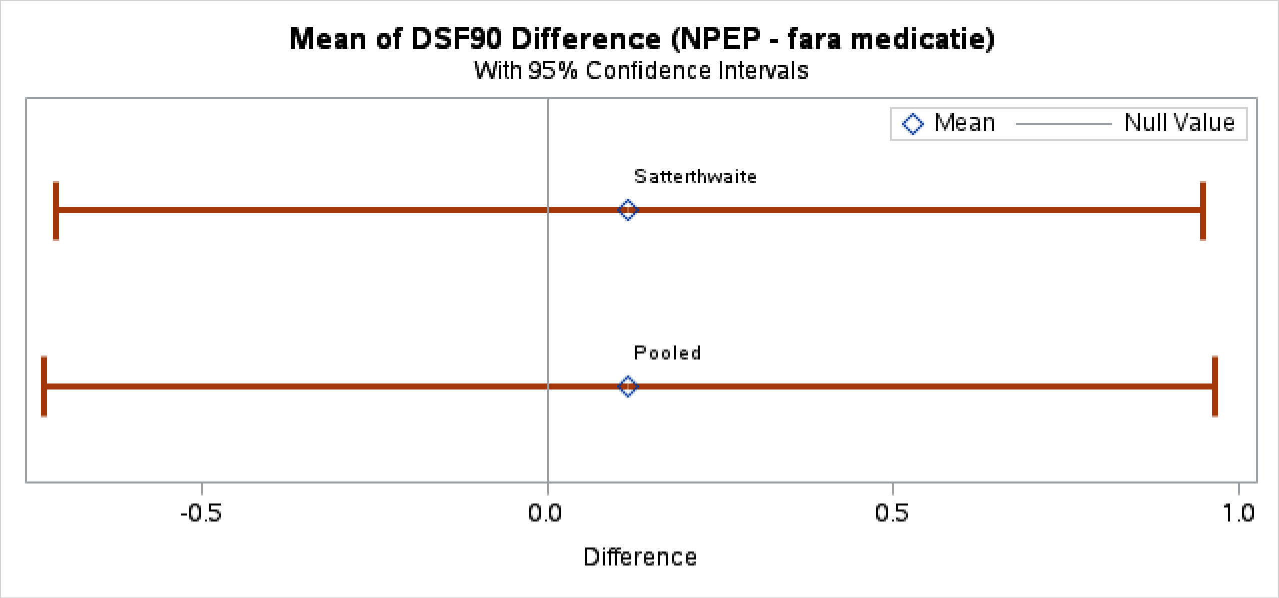

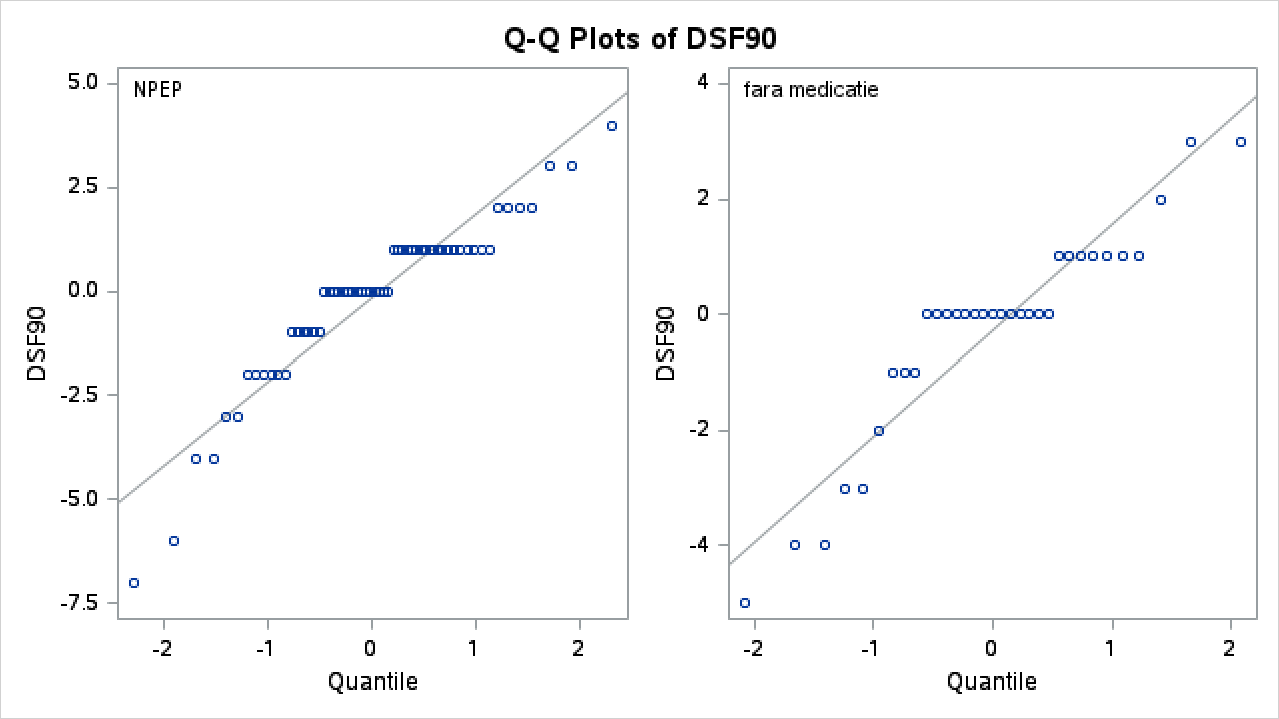

Wilcoxon Scores (Rank Sums) for Variable DSF90 Classified by Variable MEDICATIE					
MEDICATIE	N	Sum of Scores	Expected Under H0	Std Dev Under H0	Mean Score
fara medicatie	33	1449.0	1518.0	117.768299	43.909091
NPEP	58	2737.0	2668.0	117.768299	47.189655
Average scores were used for ties.					

Wilcoxon Two-Sample Test					
				t Approximation	
Statistic	Z	Pr < Z	Pr > |Z|	Pr < Z	Pr > |Z|
1449.000	-0.5817	0.2804	0.5608	0.2811	0.5623
Z includes a continuity correction of 0.5.					

Kruskal-Wallis Test		
Chi-Square	DF	Pr > ChiSq

Kruskal-Wallis Test		
Chi-Square	DF	Pr > ChiSq
0.3433	1	0.5579

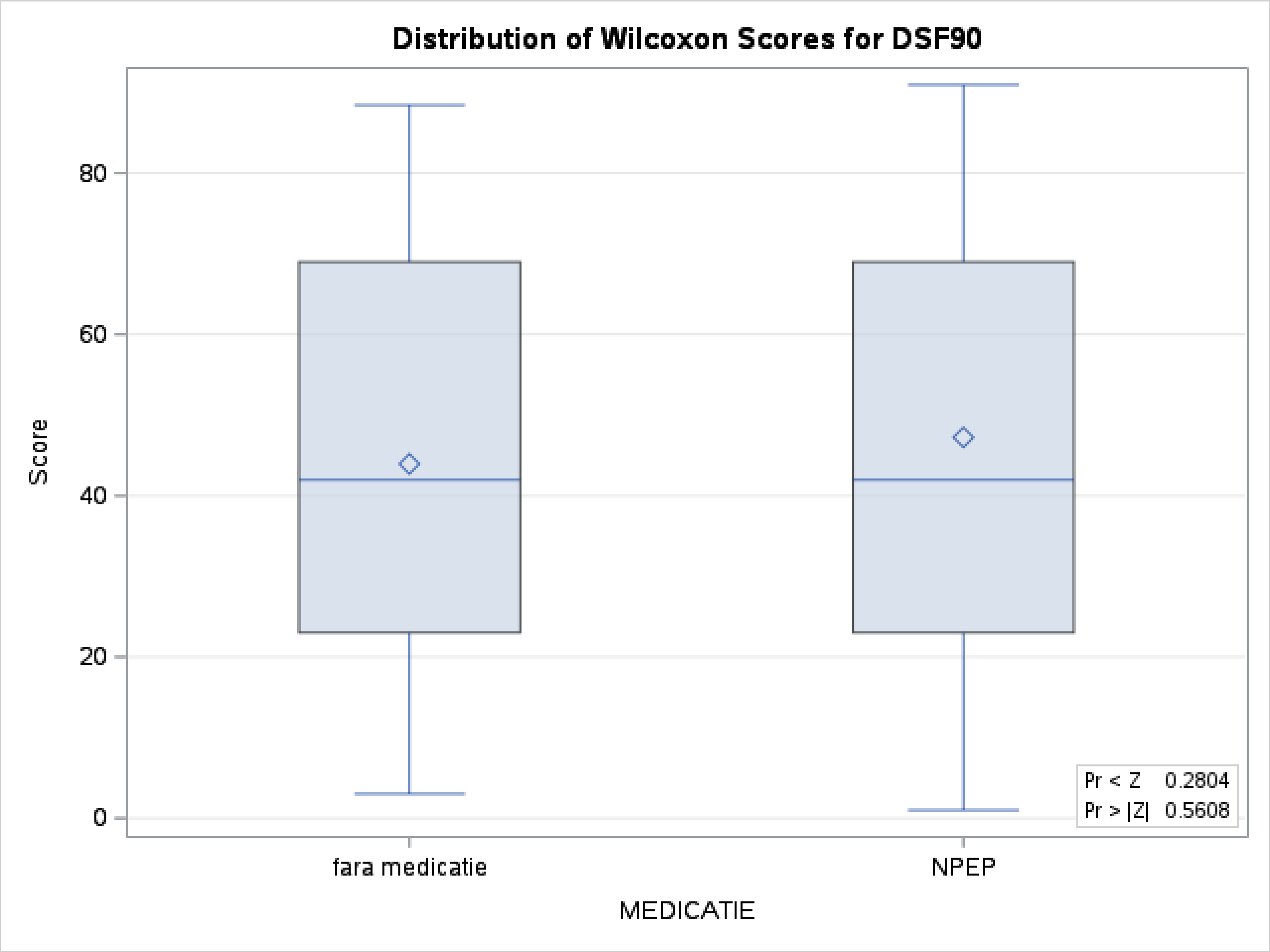

Variable: DSF360 (DSF360)
MEDICATIE = NPEP

Tests for Normality				
Test	Statistic		p Value	
Shapiro-Wilk	W	0.877768	Pr < W	<0.0001
Kolmogorov-Smirnov	D	0.167448	Pr > D	<0.0100
Cramer-von Mises	W-Sq	0.381855	Pr > W-Sq	<0.0050
Anderson-Darling	A-Sq	2.265286	Pr > A-Sq	<0.0050

Variable: DSF360 (DSF360)
MEDICATIE = fara medicatie

Tests for Normality				
Test	Statistic		p Value	
Shapiro-Wilk	W	0.857763	Pr < W	0.0005
Kolmogorov-Smirnov	D	0.193094	Pr > D	<0.0100
Cramer-von Mises	W-Sq	0.212953	Pr > W-Sq	<0.0050
Anderson-Darling	A-Sq	1.440977	Pr > A-Sq	<0.0050

Variable: DSF360 (DSF360)

MEDICATIE	Method	N	Mean	Std Dev	Std Err	Minimum	Maximum
NPEP		58	0.8621	2.2357	0.2936	-7.0000	8.0000
fara medicatie		33	0.2424	1.6399	0.2855	-4.0000	2.0000
Diff (1-2)	Pooled		0.6196	2.0416	0.4452		
Diff (1-2)	Satterthwaite		0.6196		0.4095		

MEDICATIE	Method	Mean	95% CL Mean		Std Dev	95% CL Std Dev	
NPEP		0.8621	0.2742	1.4499	2.2357	1.8900	2.7372
fara medicatie		0.2424	-0.3391	0.8239	1.6399	1.3188	2.1691
Diff (1-2)	Pooled	0.6196	-0.2649	1.5042	2.0416	1.7807	2.3927
Diff (1-2)	Satterthwaite	0.6196	-0.1948	1.4340			

Method	Variances	DF	t Value	Pr > |t|
Pooled	Equal	89	1.39	0.1674
Satterthwaite	Unequal	83.217	1.51	0.1340
Cochran	Unequal	.	1.51	0.1378

Equality of Variances				
Method	Num DF	Den DF	F Value	Pr > F
Folded F	57	32	1.86	0.0608

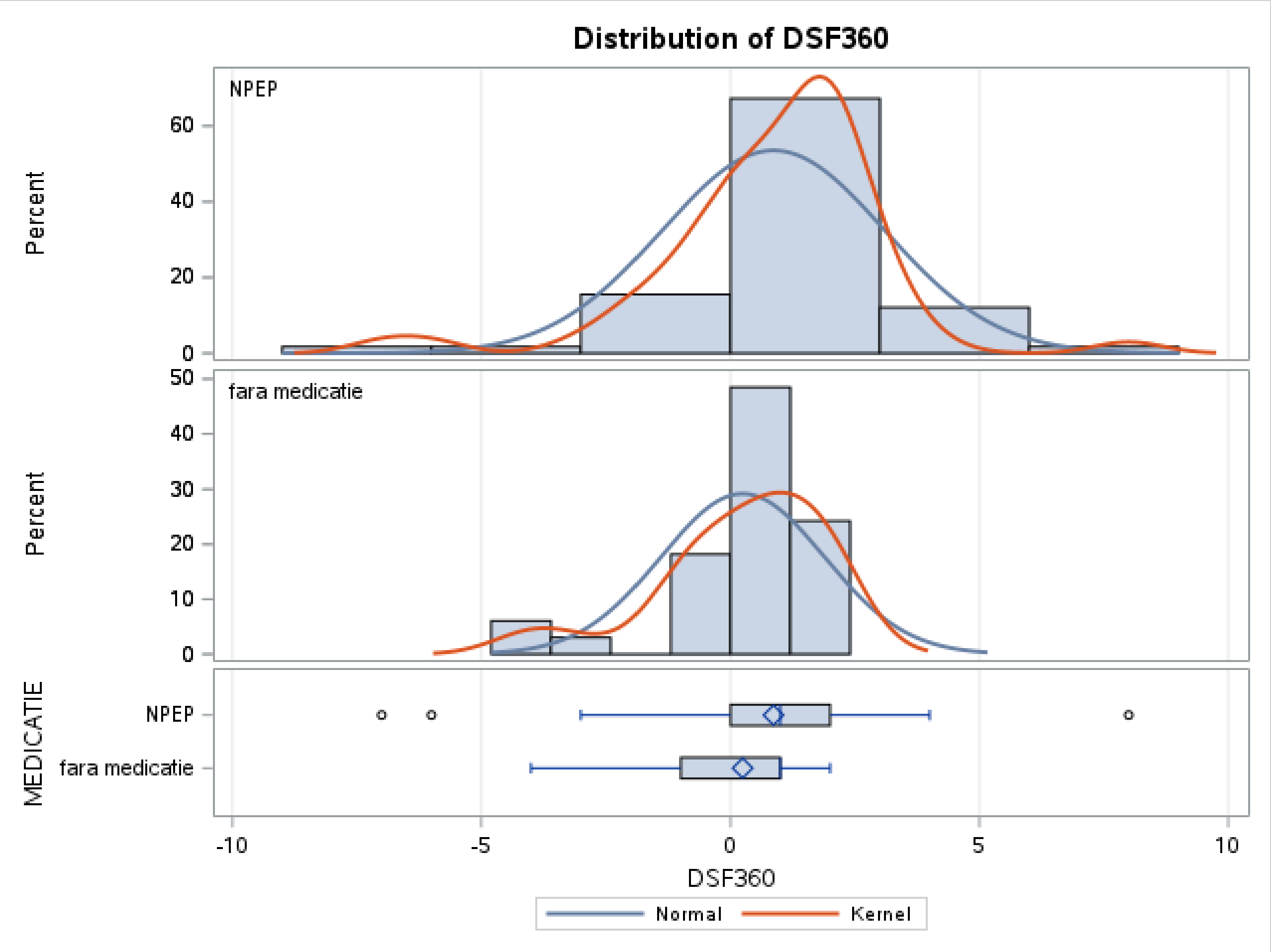

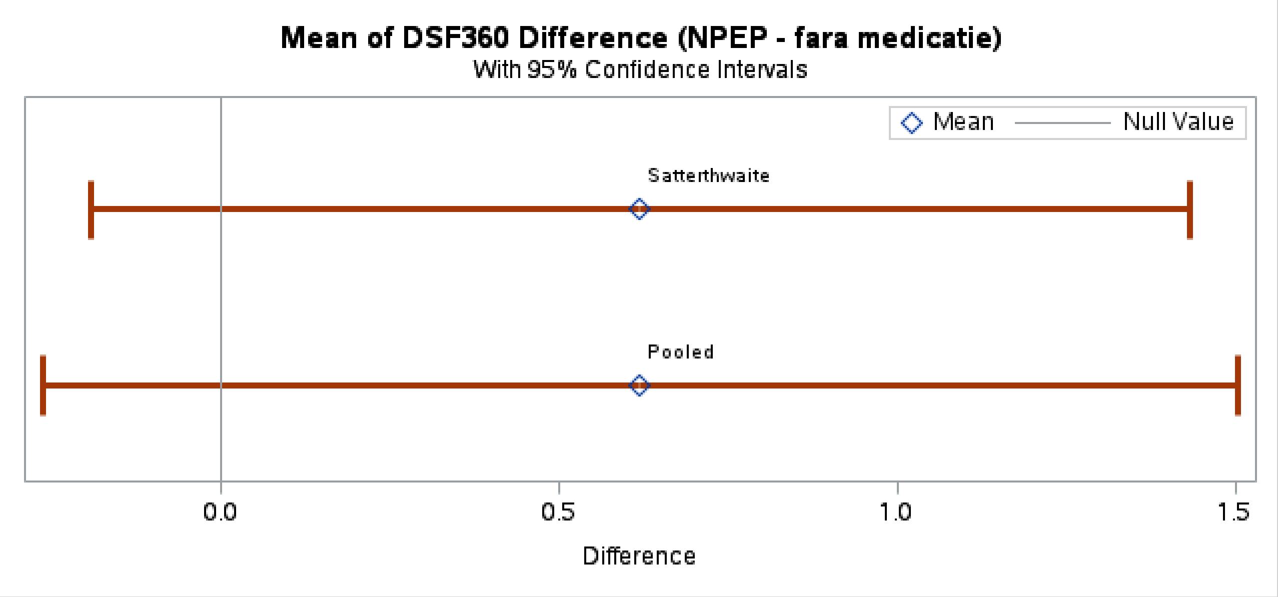

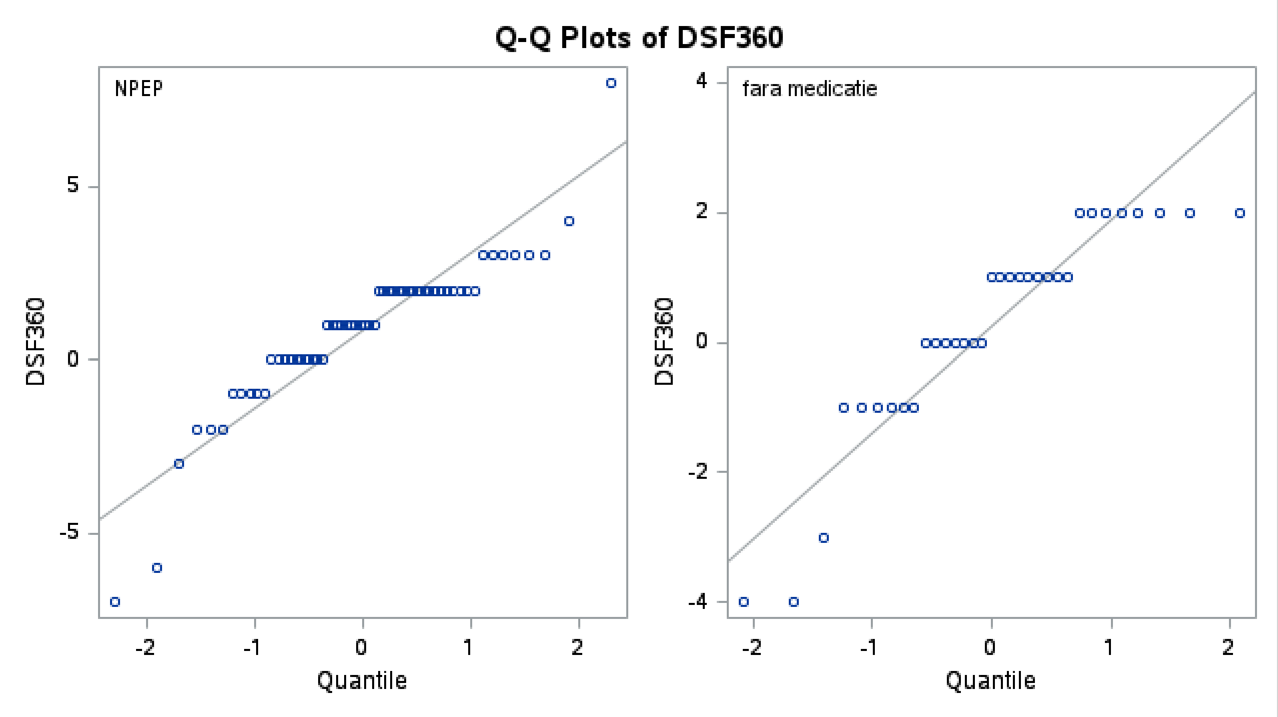

Wilcoxon Scores (Rank Sums) for Variable DSF360 Classified by Variable MEDICATIE					
MEDICATIE	N	Sum of Scores	Expected Under H0	Std Dev Under H0	Mean Score
fara medicatie	33	1297.0	1518.0	118.536732	39.303030
NPEP	58	2889.0	2668.0	118.536732	49.810345
Average scores were used for ties.					

Wilcoxon Two-Sample Test					
				t Approximation	
Statistic	Z	Pr < Z	Pr > |Z|	Pr < Z	Pr > |Z|
1297.000	-1.8602	0.0314	0.0629	0.0331	0.0661
Z includes a continuity correction of 0.5.					

Kruskal-Wallis Test		
Chi-Square	DF	Pr > ChiSq

Kruskal-Wallis Test		
Chi-Square	DF	Pr > ChiSq
3.4760	1	0.0623

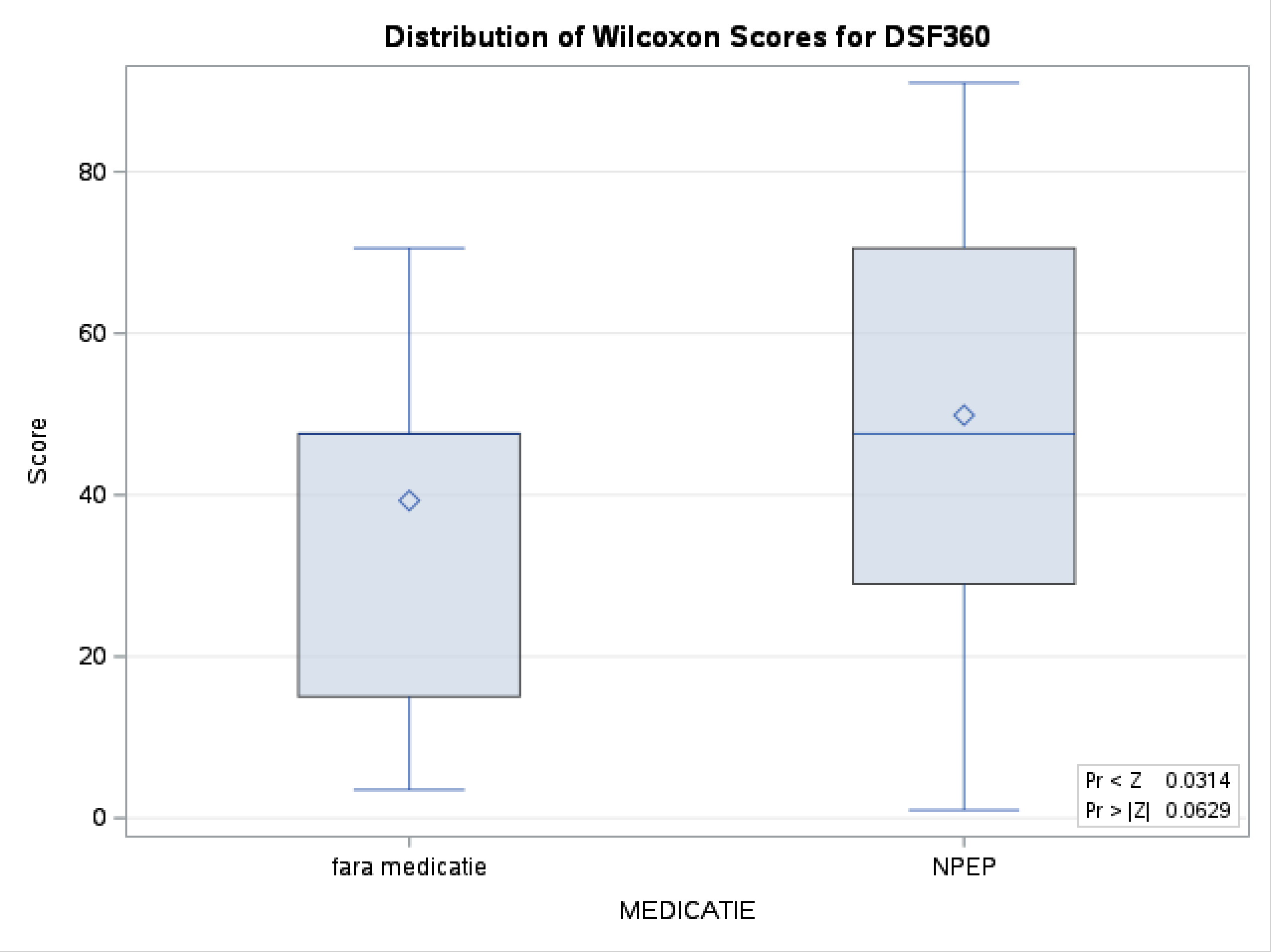

Variable: DSB90 (DSB90)
MEDICATIE = NPEP

Tests for Normality				
Test	Statistic		p Value	
Shapiro-Wilk	W	0.917862	Pr < W	0.0008
Kolmogorov-Smirnov	D	0.205909	Pr > D	<0.0100
Cramer-von Mises	W-Sq	0.335201	Pr > W-Sq	<0.0050
Anderson-Darling	A-Sq	1.832407	Pr > A-Sq	<0.0050

Variable: DSB90 (DSB90)
MEDICATIE = fara medicatie

Tests for Normality				
Test	Statistic		p Value	
Shapiro-Wilk	W	0.929825	Pr < W	0.0346
Kolmogorov-Smirnov	D	0.182704	Pr > D	<0.0100
Cramer-von Mises	W-Sq	0.187522	Pr > W-Sq	0.0073
Anderson-Darling	A-Sq	0.998555	Pr > A-Sq	0.0111

Variable: DSB90 (DSB90)

MEDICATIE	Method	N	Mean	Std Dev	Std Err	Minimum	Maximum
NPEP		58	-0.2241	1.7576	0.2308	-5.0000	3.0000
fara medicatie		33	0.0606	1.6945	0.2950	-5.0000	4.0000
Diff (1-2)	Pooled		-0.2847	1.7352	0.3784		
Diff (1-2)	Satterthwaite		-0.2847		0.3745		

MEDICATIE	Method	Mean	95% CL Mean		Std Dev	95% CL Std Dev	
NPEP		-0.2241	-0.6863	0.2380	1.7576	1.4859	2.1519
fara medicatie		0.0606	-0.5402	0.6614	1.6945	1.3627	2.2413
Diff (1-2)	Pooled	-0.2847	-1.0365	0.4670	1.7352	1.5134	2.0336
Diff (1-2)	Satterthwaite	-0.2847	-1.0320	0.4625			

Method	Variances	DF	t Value	Pr > |t|
Pooled	Equal	89	-0.75	0.4537
Satterthwaite	Unequal	68.714	-0.76	0.4497
Cochran	Unequal	.	-0.76	0.4517

Equality of Variances				
Method	Num DF	Den DF	F Value	Pr > F
Folded F	57	32	1.08	0.8393

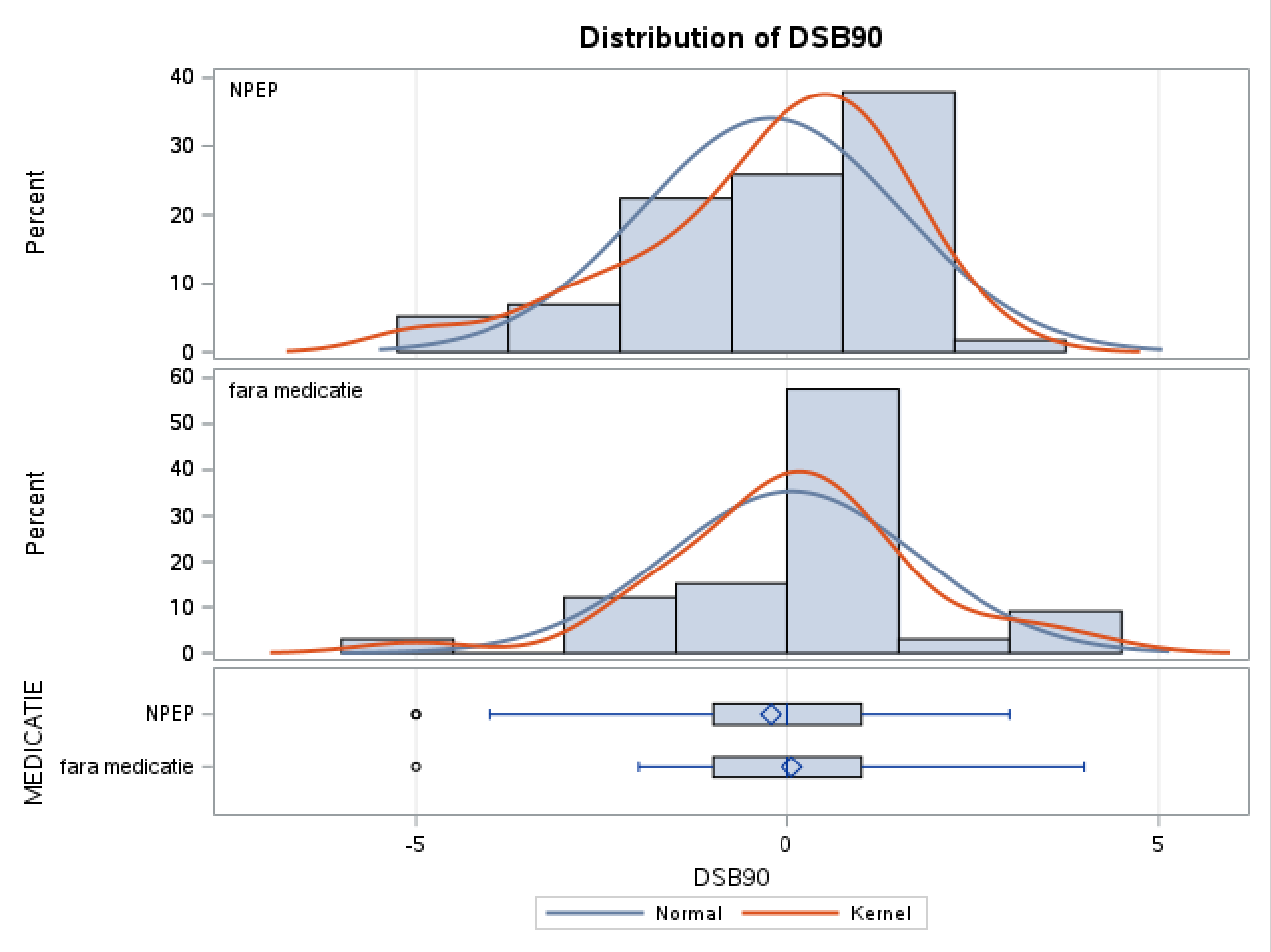

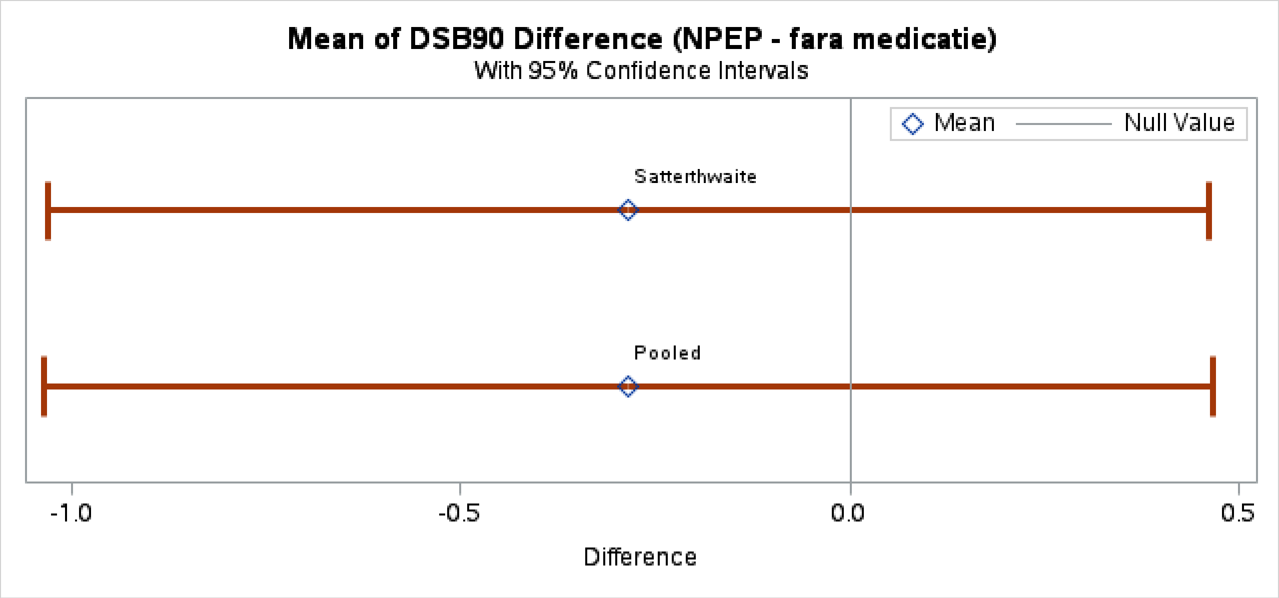

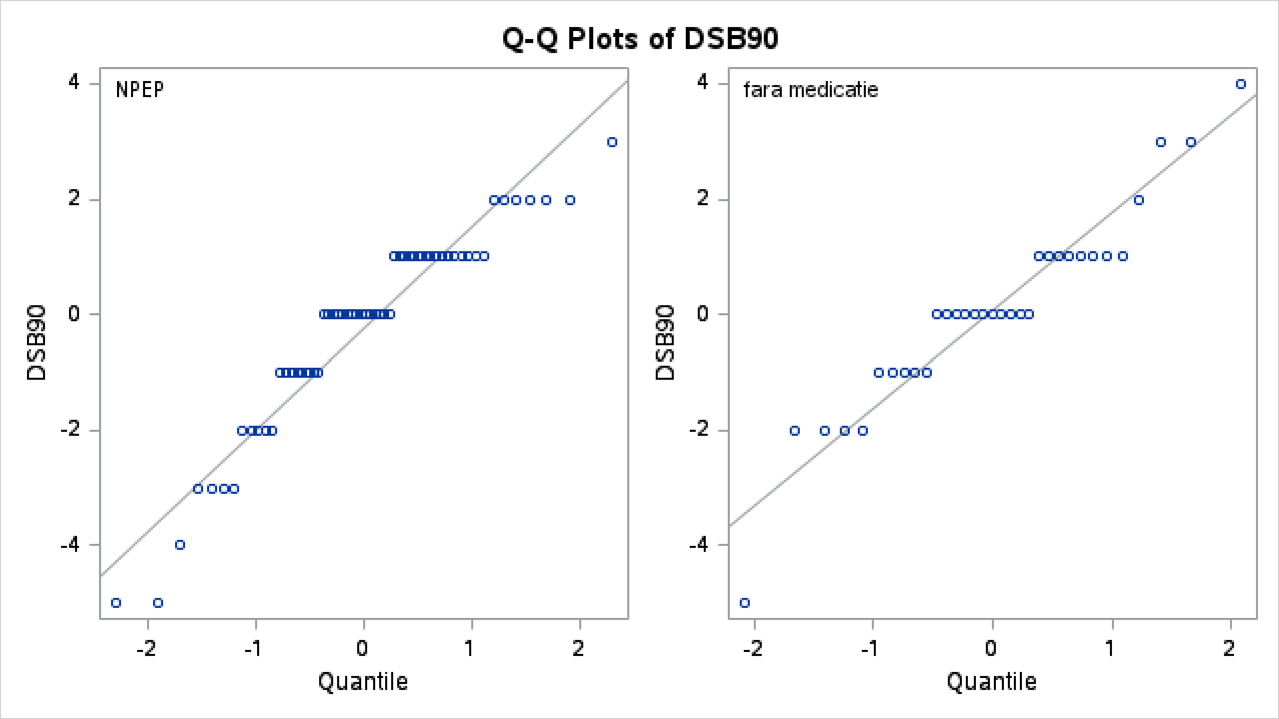

Wilcoxon Scores (Rank Sums) for Variable DSB90<br>Classified by Variable MEDICATIE					
**MEDICATIE**	**N**	**Sum of Scores**	**Expected Under H0**	**Std Dev Under H0**	**Mean Score**
fara medicatie	33	1553.50	1518.0	118.314232	47.075758
NPEP	58	2632.50	2668.0	118.314232	45.387931
Average scores were used for ties.					

Wilcoxon Two-Sample Test					
				t Approximation	
**Statistic**	**Z**	**Pr > Z**	**Pr > |Z|**	**Pr > Z**	**Pr > |Z|**
1553.500	0.2958	0.3837	0.7674	0.3840	0.7680
Z includes a continuity correction of 0.5.					

Kruskal-Wallis Test		
**Chi-Square**	**DF**	**Pr > ChiSq**

Kruskal-Wallis Test		
Chi-Square	DF	Pr > ChiSq
0.0900	1	0.7641

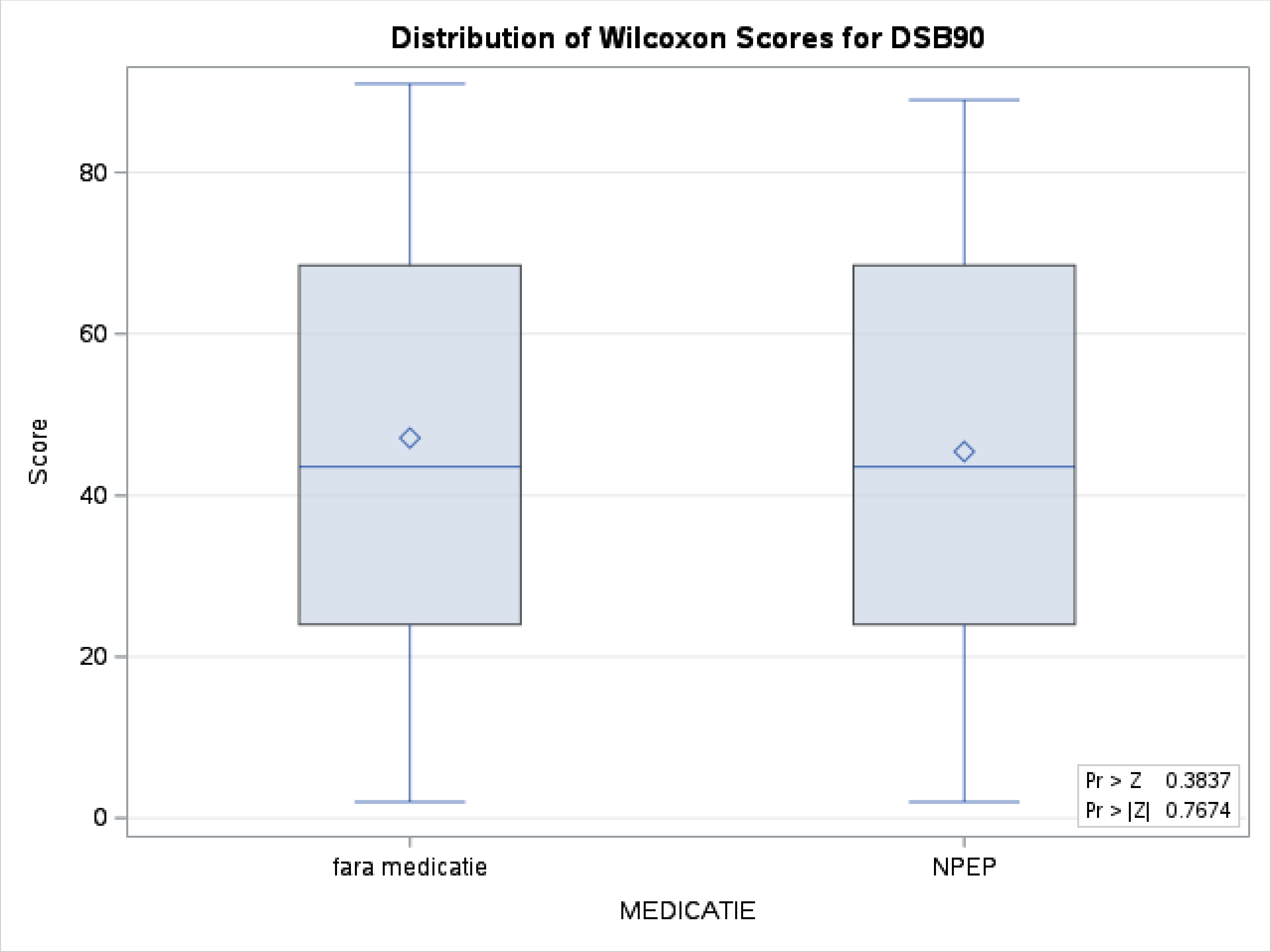

Variable: DSB360 (DSB360)
MEDICATIE = NPEP

Tests for Normality				
Test	Statistic		p Value	
Shapiro-Wilk	W	0.951226	Pr < W	0.0207
Kolmogorov-Smirnov	D	0.196057	Pr > D	<0.0100
Cramer-von Mises	W-Sq	0.282359	Pr > W-Sq	<0.0050
Anderson-Darling	A-Sq	1.384901	Pr > A-Sq	<0.0050

Variable: DSB360 (DSB360)
MEDICATIE = fara medicatie

Tests for Normality				
Test	Statistic		p Value	
Shapiro-Wilk	W	0.937605	Pr < W	0.0579
Kolmogorov-Smirnov	D	0.152422	Pr > D	0.0493
Cramer-von Mises	W-Sq	0.118975	Pr > W-Sq	0.0619
Anderson-Darling	A-Sq	0.750439	Pr > A-Sq	0.0465

Variable: DSB360 (DSB360)

MEDICATIE	Method	N	Mean	Std Dev	Std Err	Minimum	Maximum
NPEP		58	0.3448	1.7122	0.2248	-4.0000	4.0000
fara medicatie		33	-0.2727	1.5865	0.2762	-4.0000	2.0000
Diff (1-2)	Pooled		0.6176	1.6681	0.3637		
Diff (1-2)	Satterthwaite		0.6176		0.3561		

MEDICATIE	Method	Mean	95% CL Mean		Std Dev	95% CL Std Dev	
NPEP		0.3448	-0.1054	0.7950	1.7122	1.4475	2.0963
fara medicatie		-0.2727	-0.8353	0.2898	1.5865	1.2759	2.0985
Diff (1-2)	Pooled	0.6176	-0.1052	1.3403	1.6681	1.4549	1.9550
Diff (1-2)	Satterthwaite	0.6176	-0.0925	1.3276			

Method	Variances	DF	t Value	Pr > |t|
Pooled	Equal	89	1.70	0.0930
Satterthwaite	Unequal	70.968	1.73	0.0872
Cochran	Unequal	.	1.73	0.0909

Equality of Variances				
Method	Num DF	Den DF	F Value	Pr > F
Folded F	57	32	1.16	0.6511

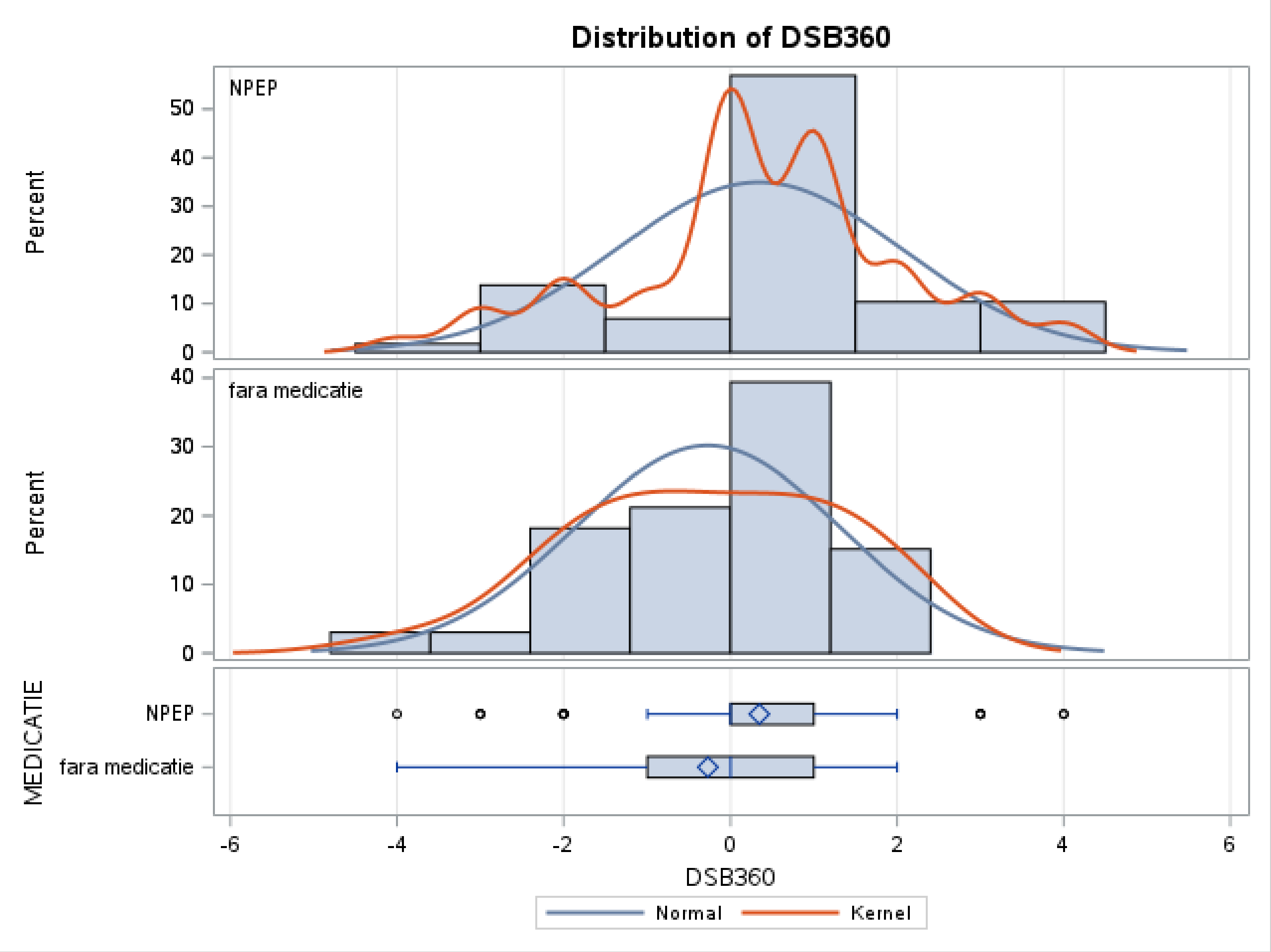

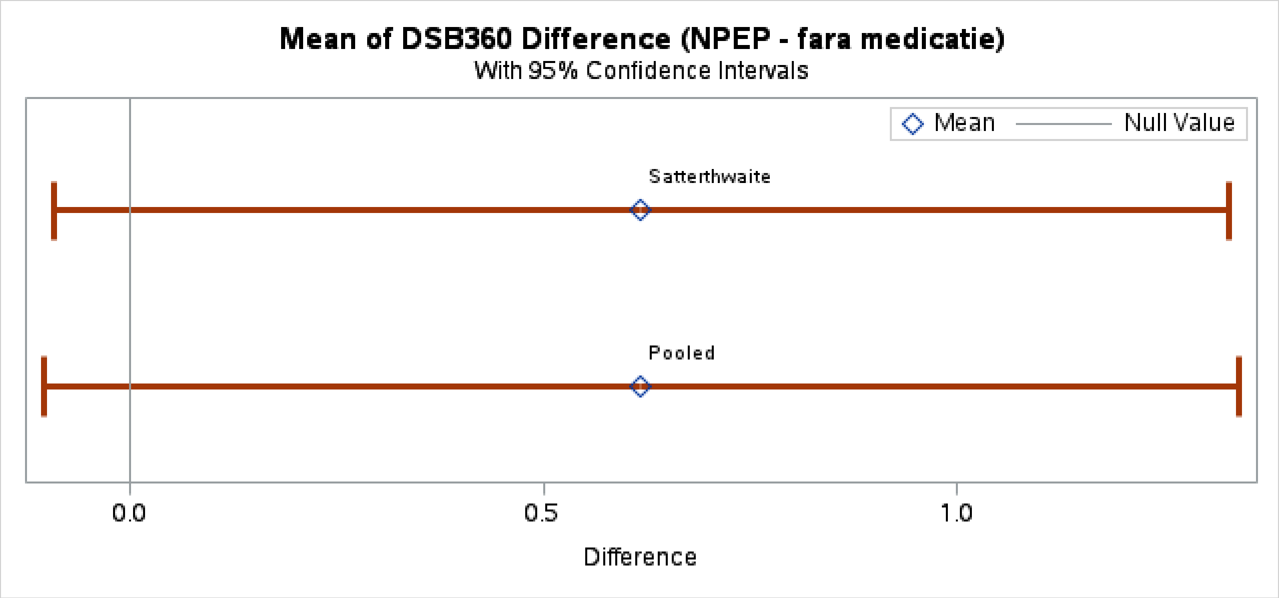

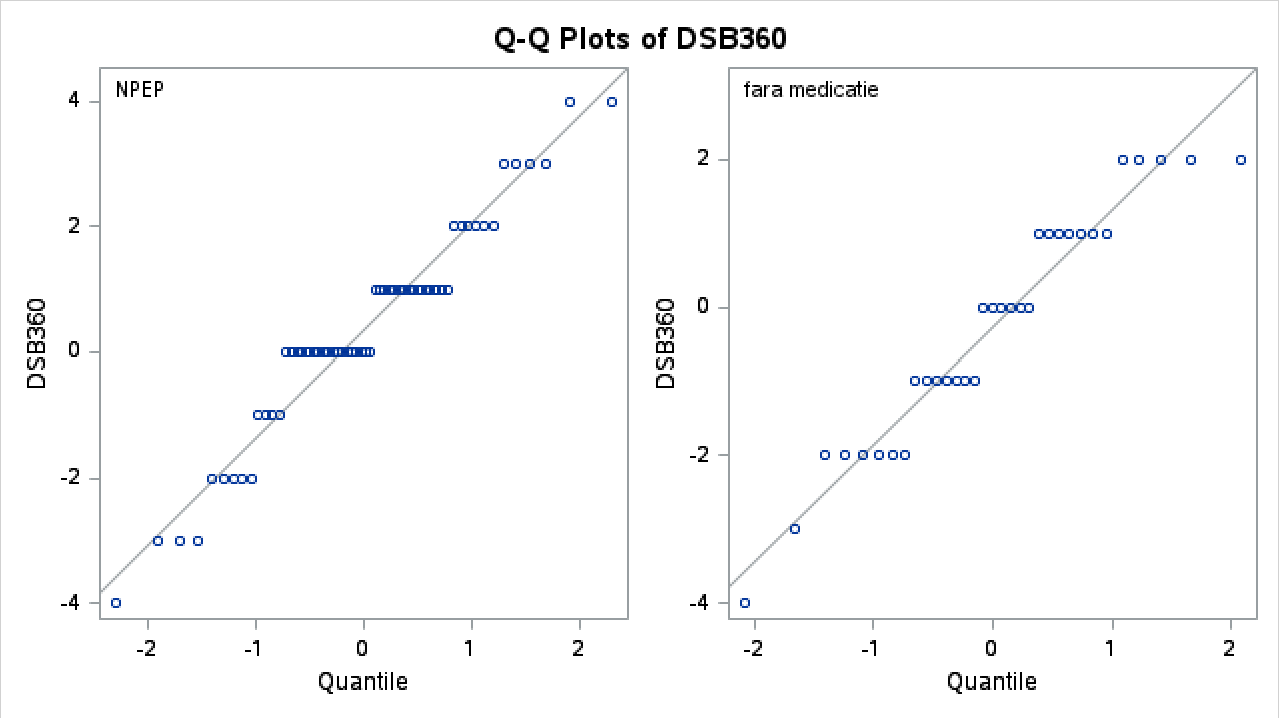

Wilcoxon Scores (Rank Sums) for Variable DSB360 Classified by Variable MEDICATIE					
MEDICATIE	N	Sum of Scores	Expected Under H0	Std Dev Under H0	Mean Score
fara medicatie	33	1326.50	1518.0	118.821769	40.196970
NPEP	58	2859.50	2668.0	118.821769	49.301724
Average scores were used for ties.					

Wilcoxon Two-Sample Test					
				t Approximation	
Statistic	Z	Pr < Z	Pr > |Z|	Pr < Z	Pr > |Z|
1326.500	-1.6074	0.0540	0.1080	0.0557	0.1115
Z includes a continuity correction of 0.5.					

Kruskal-Wallis Test		
Chi-Square	DF	Pr > ChiSq

Kruskal-Wallis Test		
Chi-Square	DF	Pr > ChiSq
2.5974	1	0.1070

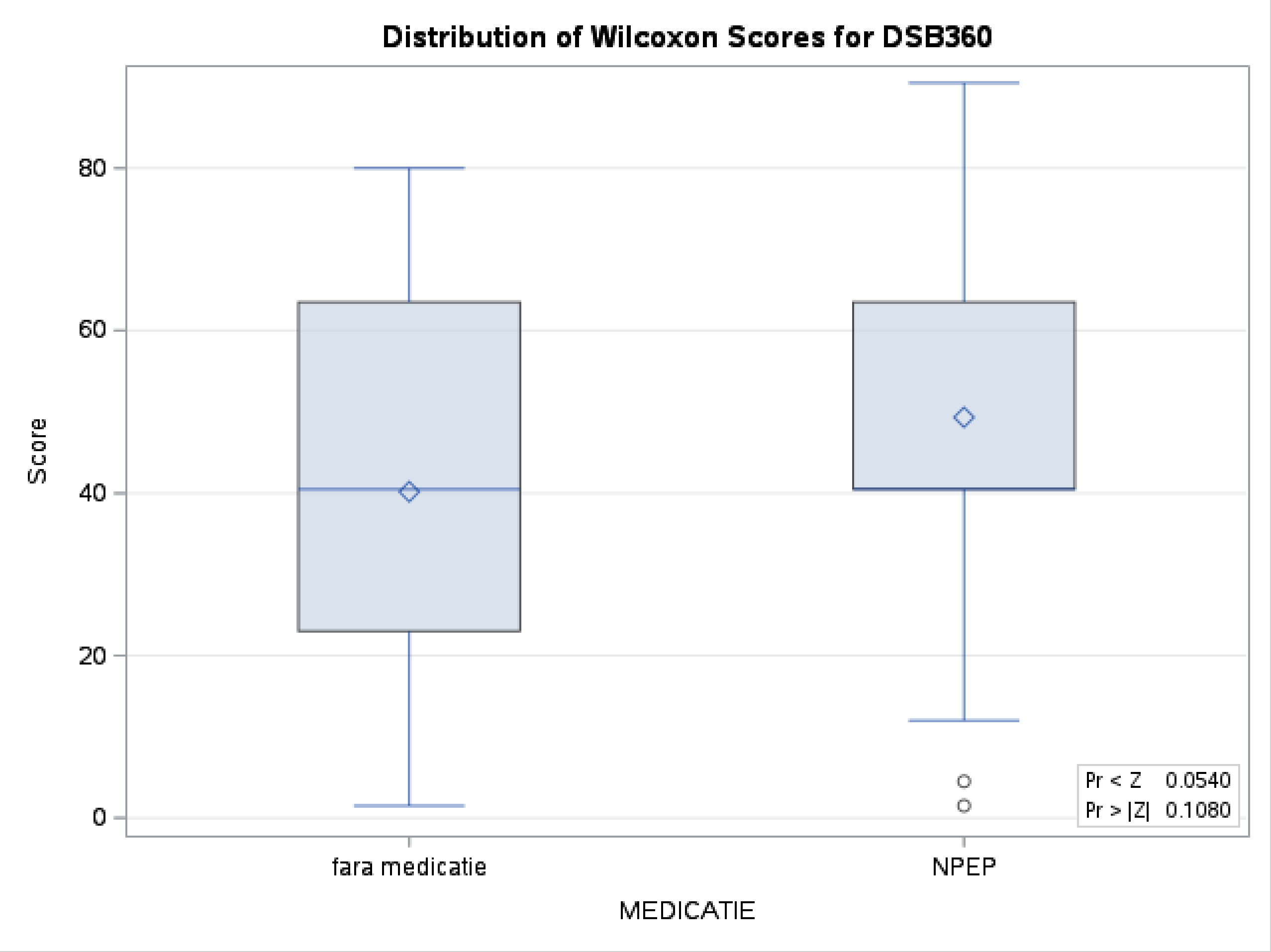

Variable: DSC90 (DSC90)
MEDICATIE = NPEP

Tests for Normality				
Test	Statistic		p Value	
Shapiro-Wilk	W	0.99224	Pr < W	0.9723
Kolmogorov-Smirnov	D	0.082214	Pr > D	>0.1500
Cramer-von Mises	W-Sq	0.040648	Pr > W-Sq	>0.2500
Anderson-Darling	A-Sq	0.218182	Pr > A-Sq	>0.2500

Variable: DSC90 (DSC90)
MEDICATIE = fara medicatie

Tests for Normality				
Test	Statistic		p Value	
Shapiro-Wilk	W	0.775412	Pr < W	<0.0001
Kolmogorov-Smirnov	D	0.199264	Pr > D	<0.0100
Cramer-von Mises	W-Sq	0.423181	Pr > W-Sq	<0.0050
Anderson-Darling	A-Sq	2.384069	Pr > A-Sq	<0.0050

Variable: DSC90 (DSC90)

MEDICATIE	Method	N	Mean	Std Dev	Std Err	Minimum	Maximum
NPEP		58	4.0690	6.8898	0.9047	-13.0000	21.0000
fara medicatie		33	6.3939	12.0207	2.0925	-25.0000	55.0000
Diff (1-2)	Pooled		-2.3250	9.0750	1.9788		
Diff (1-2)	Satterthwaite		-2.3250		2.2797		

MEDICATIE	Method	Mean	95% CL Mean		Std Dev	95% CL Std Dev	
NPEP		4.0690	2.2574	5.8805	6.8898	5.8247	8.4352
fara medicatie		6.3939	2.1316	10.6563	12.0207	9.6669	15.8996
Diff (1-2)	Pooled	-2.3250	-6.2567	1.6068	9.0750	7.9153	10.6359
Diff (1-2)	Satterthwaite	-2.3250	-6.9188	2.2689			

Method	Variances	DF	t Value	Pr > |t|
Pooled	Equal	89	-1.17	0.2431
Satterthwaite	Unequal	44.213	-1.02	0.3133
Cochran	Unequal	.	-1.02	0.3149

Equality of Variances				
Method	Num DF	Den DF	F Value	Pr > F
Folded F	32	57	3.04	0.0002

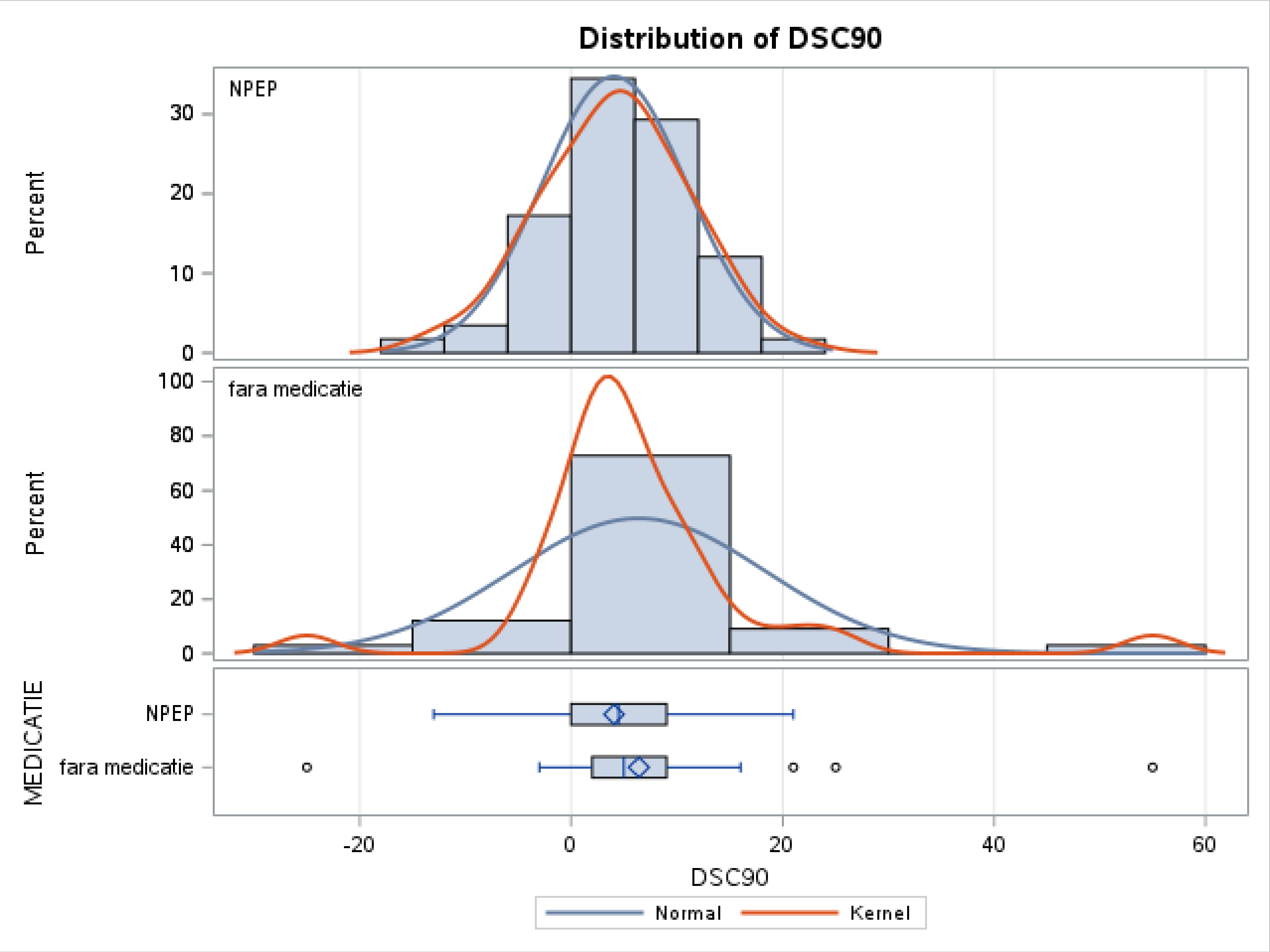

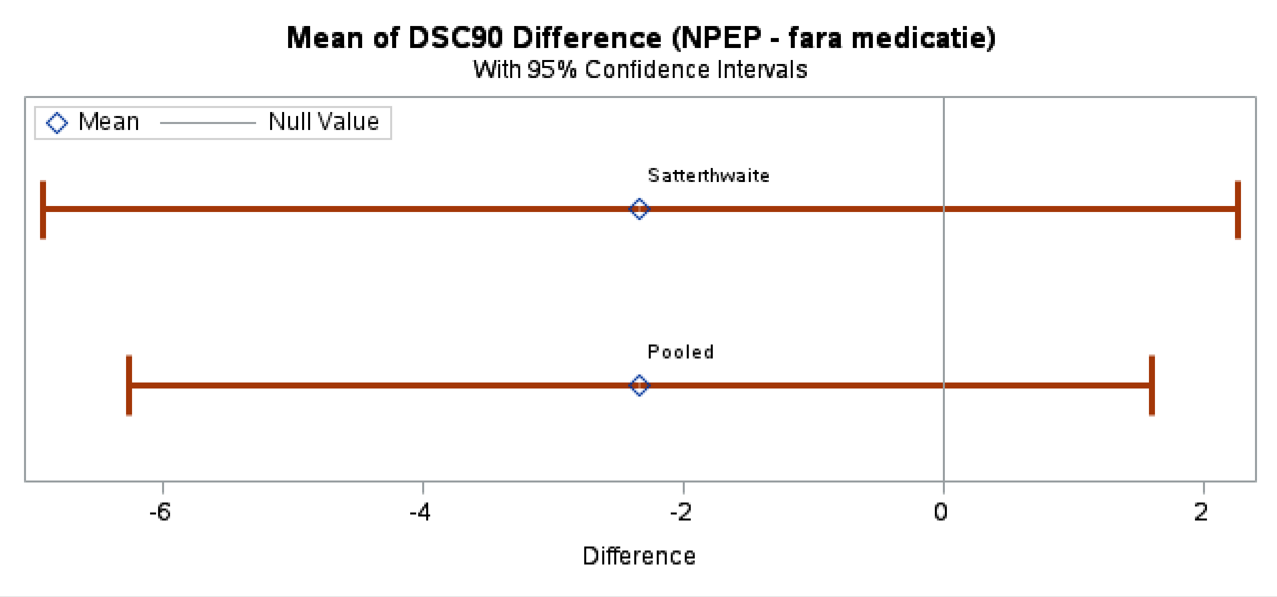

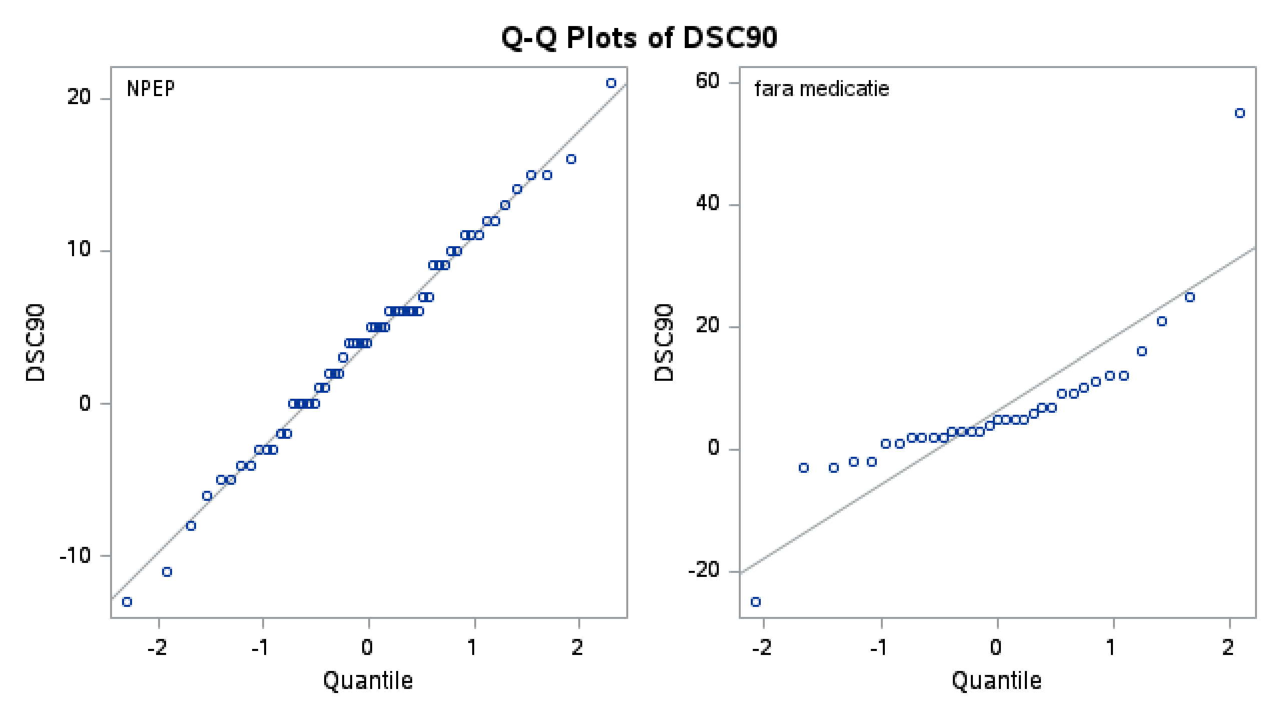

Wilcoxon Scores (Rank Sums) for Variable DSC90<br>Classified by Variable MEDICATIE					
MEDICATIE	N	Sum of Scores	Expected Under H0	Std Dev Under H0	Mean Score
fara medicatie	33	1604.50	1518.0	120.944176	48.621212
NPEP	58	2581.50	2668.0	120.944176	44.508621
Average scores were used for ties.					

Wilcoxon Two-Sample Test					
				t Approximation	
Statistic	Z	Pr > Z	Pr > |Z|	Pr > Z	Pr > |Z|
1604.500	0.7111	0.2385	0.4770	0.2394	0.4789
Z includes a continuity correction of 0.5.					

Kruskal-Wallis Test		
Chi-Square	DF	Pr > ChiSq

Kruskal-Wallis Test		
Chi-Square	DF	Pr > ChiSq
0.5115	1	0.4745

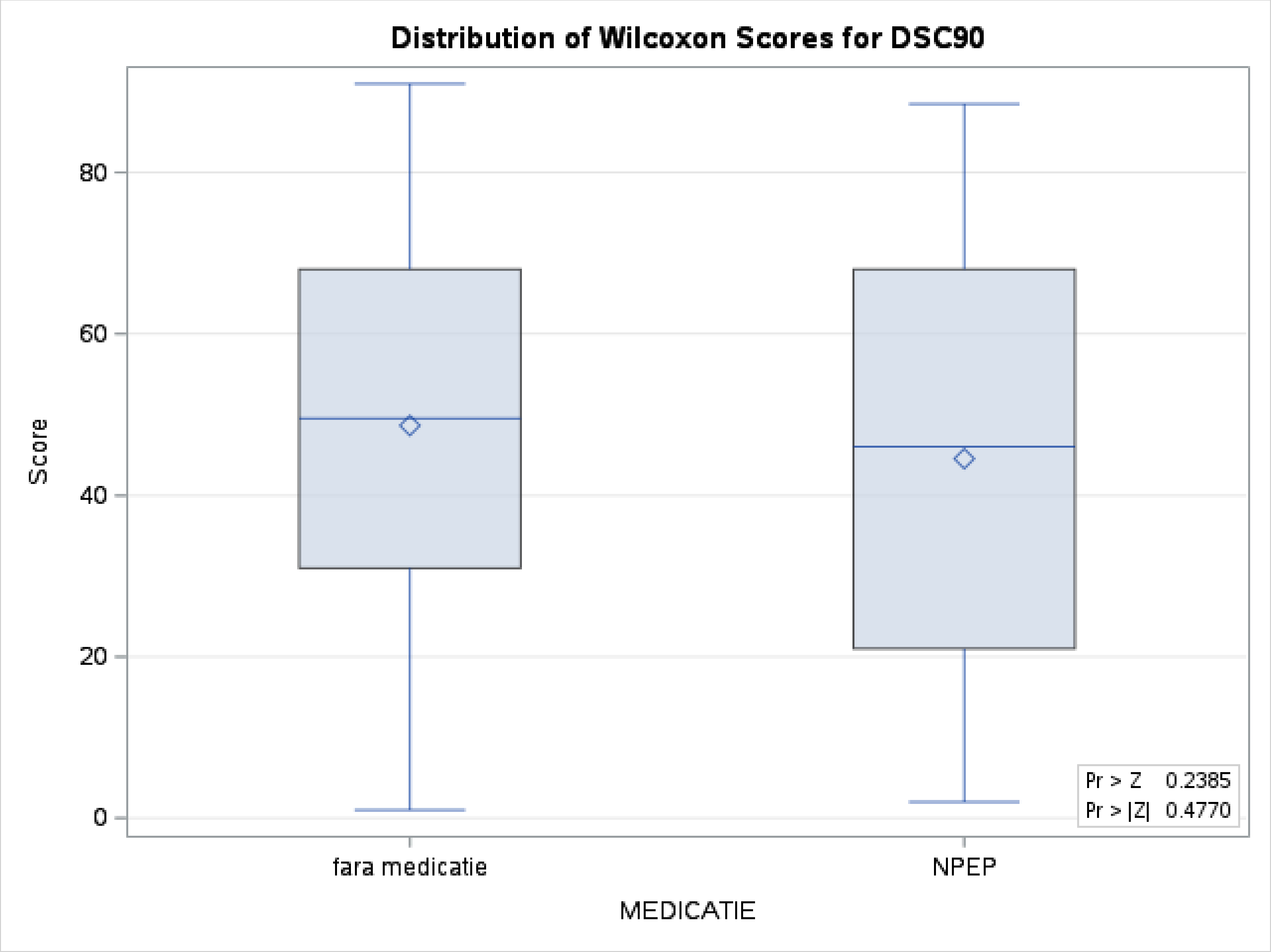

Variable: DSC360 (DSC360)
MEDICATIE = NPEP

Tests for Normality				
Test	Statistic		p Value	
Shapiro-Wilk	W	0.979873	Pr < W	0.4463
Kolmogorov-Smirnov	D	0.091419	Pr > D	>0.1500
Cramer-von Mises	W-Sq	0.059806	Pr > W-Sq	>0.2500
Anderson-Darling	A-Sq	0.39899	Pr > A-Sq	>0.2500

Variable: DSC360 (DSC360)
MEDICATIE = fara medicatie

Tests for Normality				
Test	Statistic		p Value	
Shapiro-Wilk	W	0.899283	Pr < W	0.0051
Kolmogorov-Smirnov	D	0.195473	Pr > D	<0.0100
Cramer-von Mises	W-Sq	0.280401	Pr > W-Sq	<0.0050
Anderson-Darling	A-Sq	1.483096	Pr > A-Sq	<0.0050

Variable: DSC360 (DSC360)

MEDICATIE	Method	N	Mean	Std Dev	Std Err	Minimum	Maximum
NPEP		58	6.3621	6.1035	0.8014	-8.0000	22.0000
fara medicatie		33	2.0909	8.9455	1.5572	-19.0000	27.0000
Diff (1-2)	Pooled		4.2712	7.2547	1.5819		
Diff (1-2)	Satterthwaite		4.2712		1.7513		

MEDICATIE	Method	Mean	95% CL Mean		Std Dev	95% CL Std Dev	
NPEP		6.3621	4.7572	7.9669	6.1035	5.1599	7.4726
fara medicatie		2.0909	-1.0810	5.2629	8.9455	7.1939	11.8322
Diff (1-2)	Pooled	4.2712	1.1280	7.4143	7.2547	6.3276	8.5025
Diff (1-2)	Satterthwaite	4.2712	0.7522	7.7902			

Method	Variances	DF	t Value	Pr > |t|
Pooled	Equal	89	2.70	0.0083
Satterthwaite	Unequal	49.257	2.44	0.0184
Cochran	Unequal	.	2.44	0.0199

Equality of Variances				
Method	Num DF	Den DF	F Value	Pr > F
Folded F	32	57	2.15	0.0116

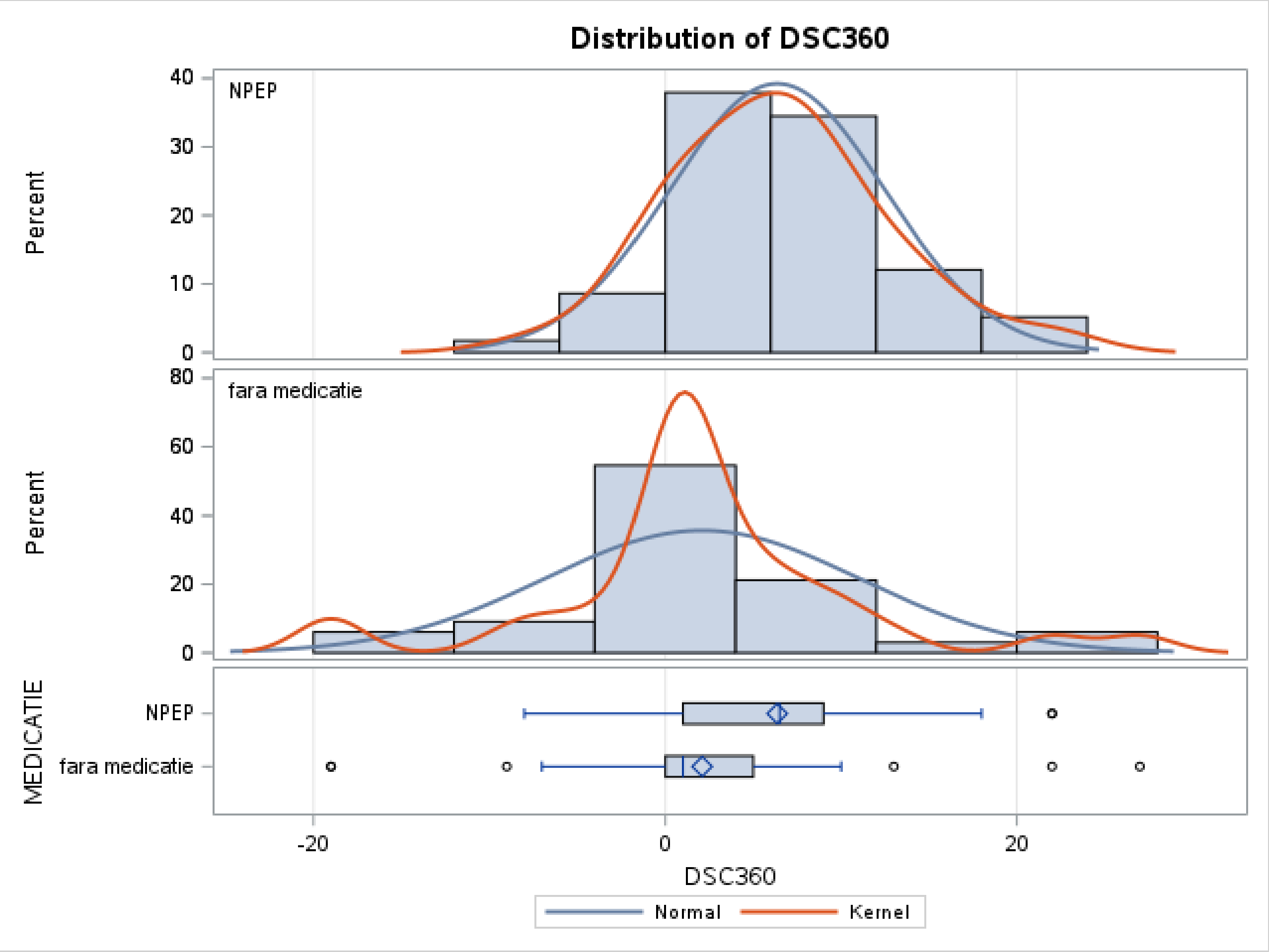

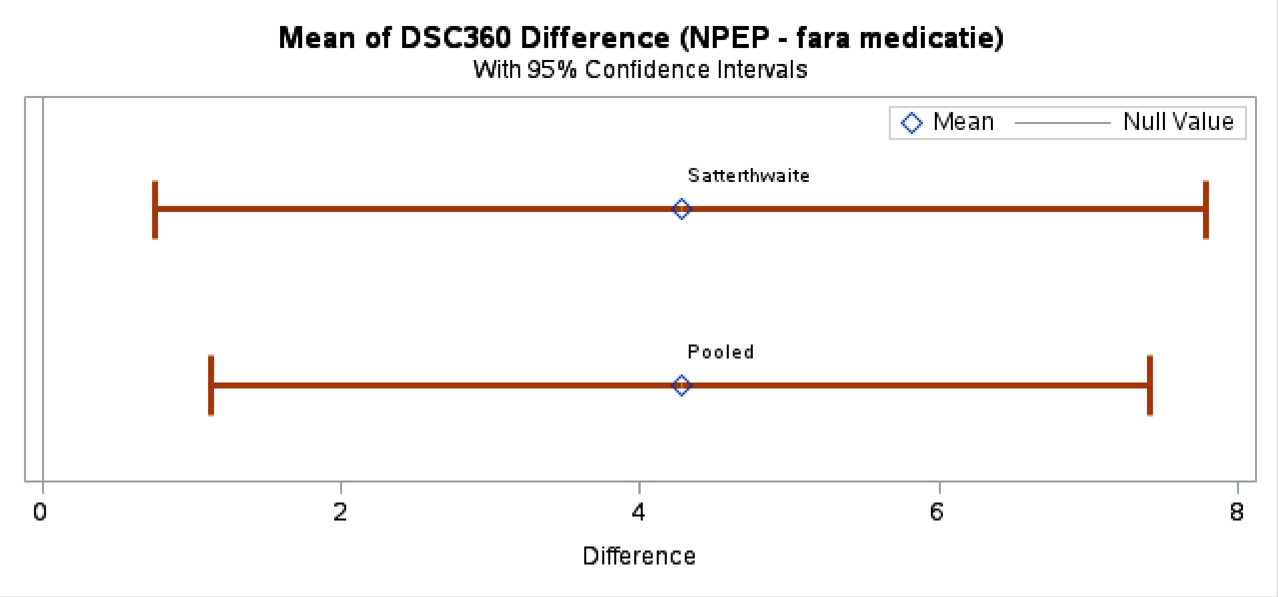

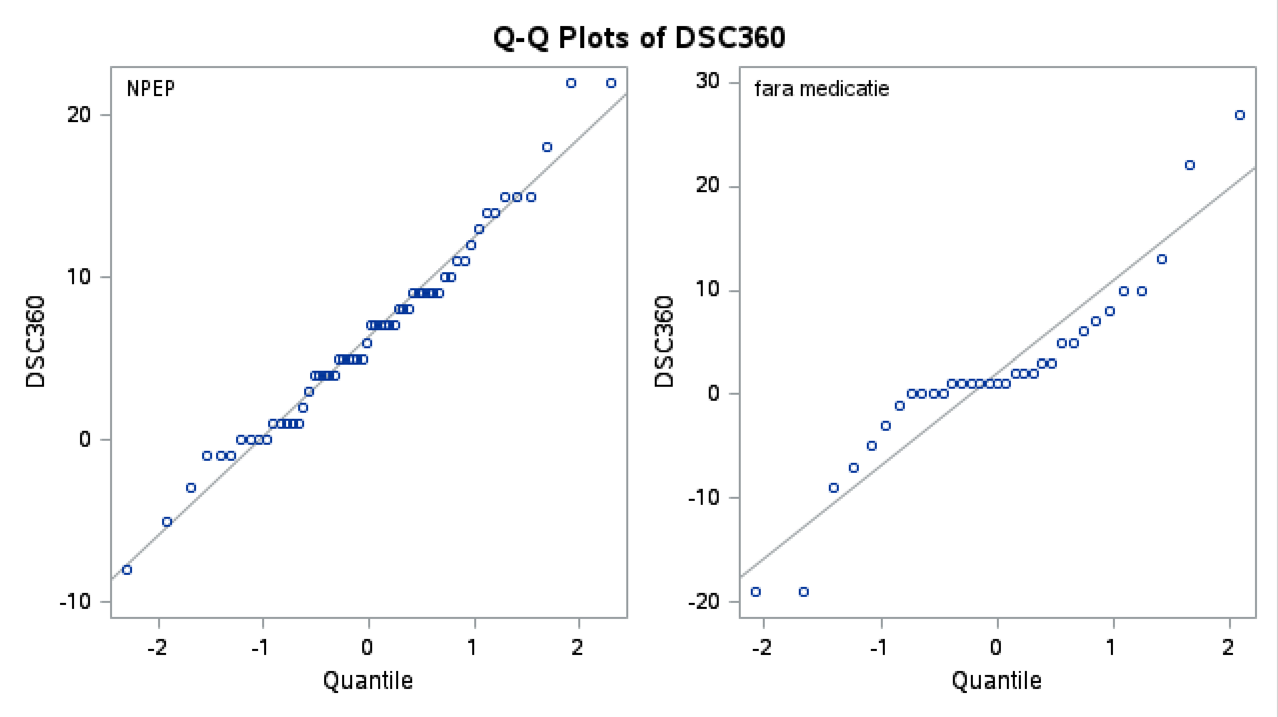

Wilcoxon Scores (Rank Sums) for Variable DSC360 Classified by Variable MEDICATIE					
MEDICATIE	N	Sum of Scores	Expected Under H0	Std Dev Under H0	Mean Score
fara medicatie	33	1171.0	1518.0	120.834952	35.484848
NPEP	58	3015.0	2668.0	120.834952	51.982759
Average scores were used for ties.					

Wilcoxon Two-Sample Test					
				t Approximation	
Statistic	Z	Pr < Z	Pr > |Z|	Pr < Z	Pr > |Z|
1171.000	-2.8675	0.0021	0.0041	0.0026	0.0052
Z includes a continuity correction of 0.5.					

Kruskal-Wallis Test		
Chi-Square	DF	Pr > ChiSq

Kruskal-Wallis Test		
Chi-Square	DF	Pr > ChiSq
8.2466	1	0.0041

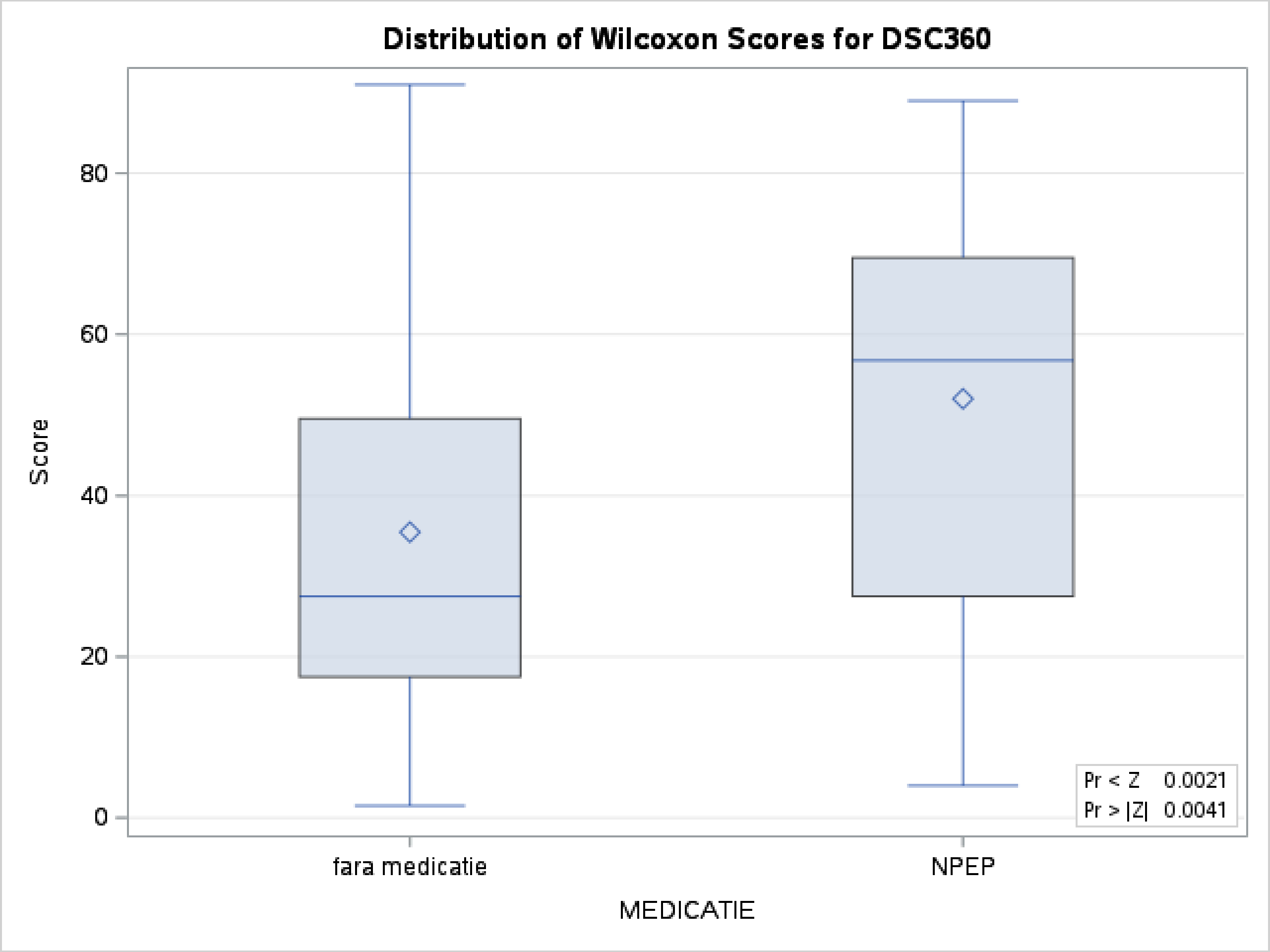

Variable: SS90 (SS90)
MEDICATIE = NPEP

Tests for Normality				
Test	Statistic		p Value	
Shapiro-Wilk	W	0.964817	Pr < W	0.0907
Kolmogorov-Smirnov	D	0.122555	Pr > D	0.0288
Cramer-von Mises	W-Sq	0.077166	Pr > W-Sq	0.2275
Anderson-Darling	A-Sq	0.450606	Pr > A-Sq	>0.2500

Variable: SS90 (SS90)
MEDICATIE = fara medicatie

Tests for Normality				
Test	Statistic		p Value	
Shapiro-Wilk	W	0.791292	Pr < W	<0.0001
Kolmogorov-Smirnov	D	0.190088	Pr > D	<0.0100
Cramer-von Mises	W-Sq	0.243471	Pr > W-Sq	<0.0050
Anderson-Darling	A-Sq	1.52164	Pr > A-Sq	<0.0050

Variable: SS90 (SS90)

MEDICATIE	Method	N	Mean	Std Dev	Std Err	Minimum	Maximum
NPEP		58	2.2241	4.0481	0.5315	-12.0000	10.0000
fara medicatie		33	1.6364	3.8957	0.6781	-3.0000	18.0000
Diff (1-2)	Pooled		0.5878	3.9940	0.8709		
Diff (1-2)	Satterthwaite		0.5878		0.8616		

MEDICATIE	Method	Mean	95% CL Mean		Std Dev	95% CL Std Dev	
NPEP		2.2241	1.1597	3.2885	4.0481	3.4223	4.9562
fara medicatie		1.6364	0.2550	3.0177	3.8957	3.1328	5.1528
Diff (1-2)	Pooled	0.5878	-1.1426	2.3182	3.9940	3.4836	4.6810
Diff (1-2)	Satterthwaite	0.5878	-1.1312	2.3068			

Method	Variances	DF	t Value	Pr > |t|
Pooled	Equal	89	0.67	0.5015
Satterthwaite	Unequal	68.816	0.68	0.4974
Cochran	Unequal	.	0.68	0.4992

Equality of Variances				
Method	Num DF	Den DF	F Value	Pr > F
Folded F	57	32	1.08	0.8304

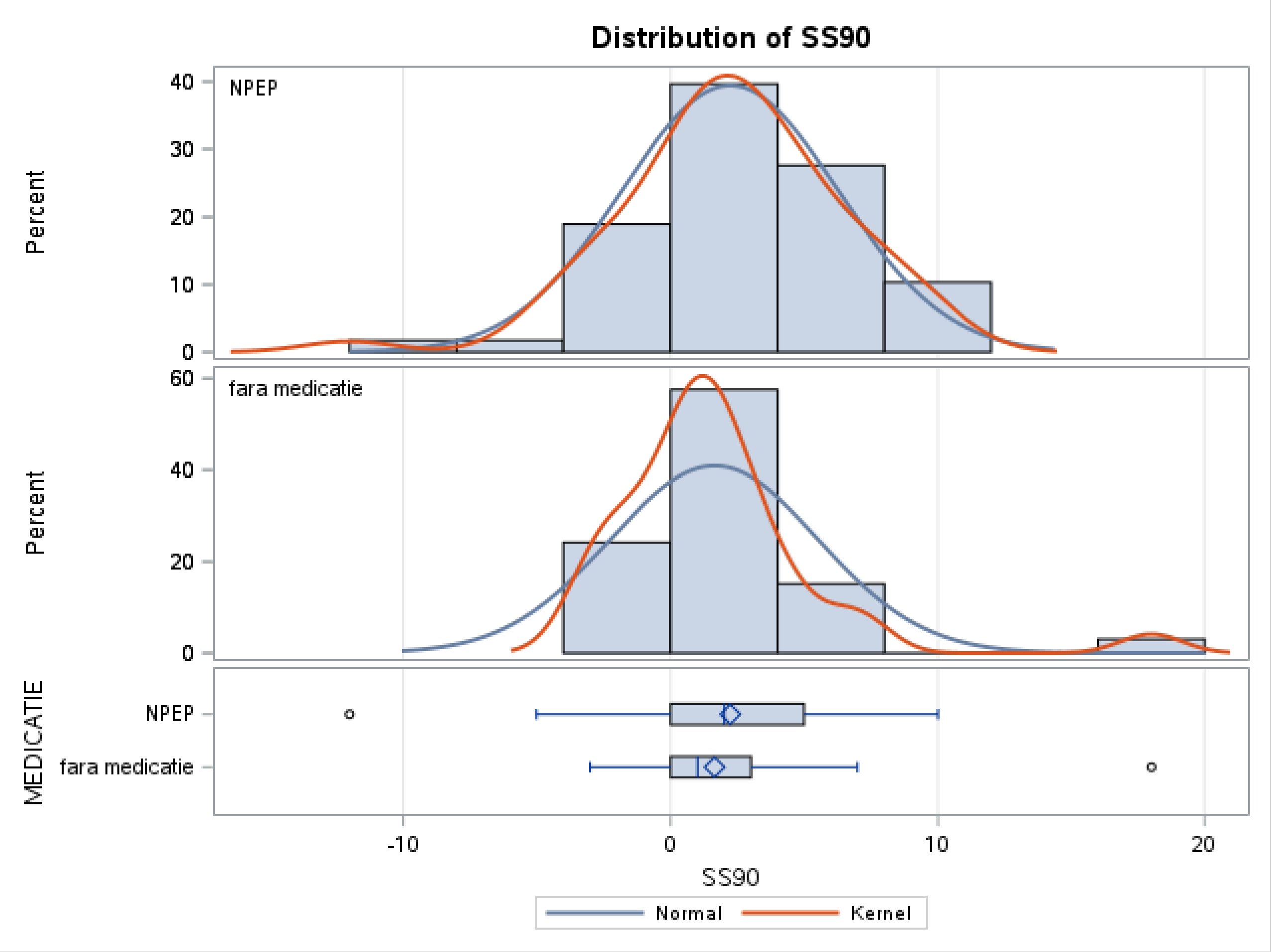

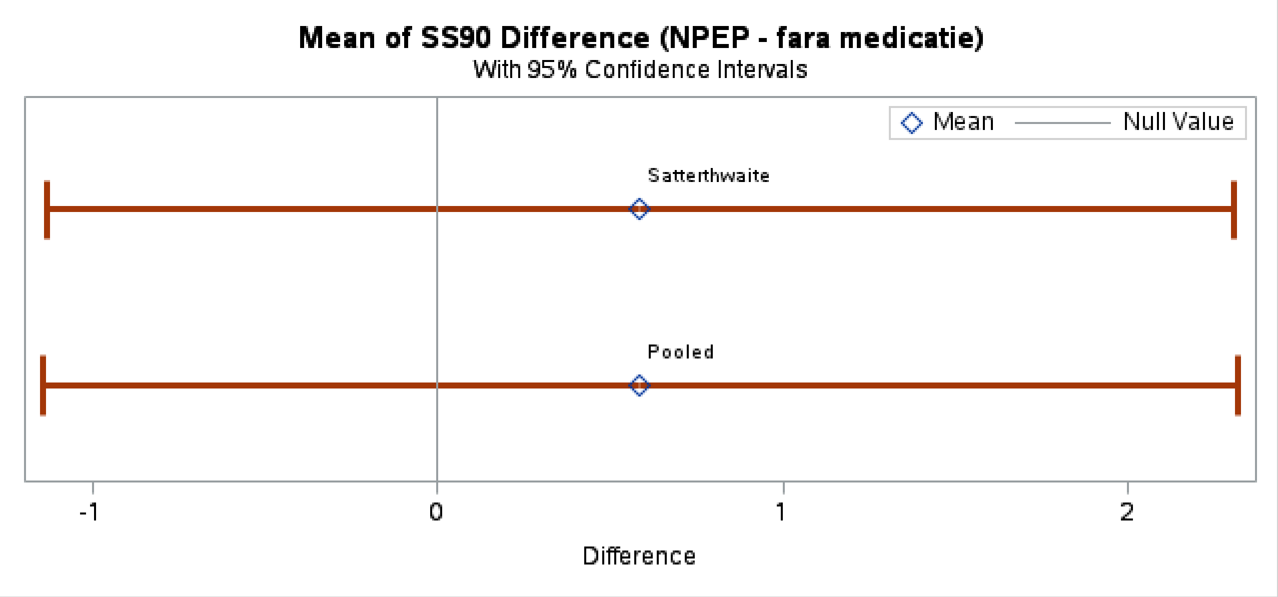

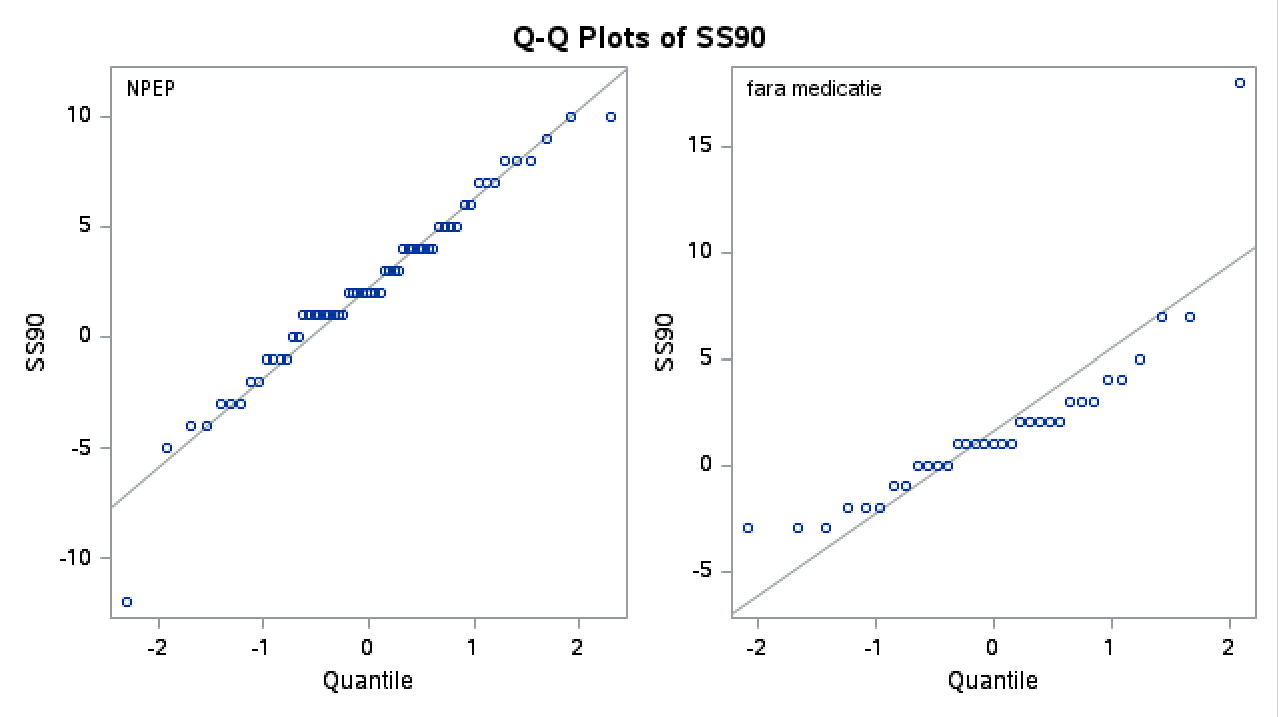

Wilcoxon Scores (Rank Sums) for Variable SS90 Classified by Variable MEDICATIE					
MEDICATIE	N	Sum of Scores	Expected Under H0	Std Dev Under H0	Mean Score
fara medicatie	33	1341.0	1518.0	120.463044	40.636364
NPEP	58	2845.0	2668.0	120.463044	49.051724
Average scores were used for ties.					

Wilcoxon Two-Sample Test					
				t Approximation	
Statistic	Z	Pr < Z	Pr > |Z|	Pr < Z	Pr > |Z|
1341.000	-1.4652	0.0714	0.1429	0.0732	0.1464
Z includes a continuity correction of 0.5.					

Kruskal-Wallis Test		
Chi-Square	DF	Pr > ChiSq

Kruskal-Wallis Test		
Chi-Square	DF	Pr > ChiSq
2.1589	1	0.1417

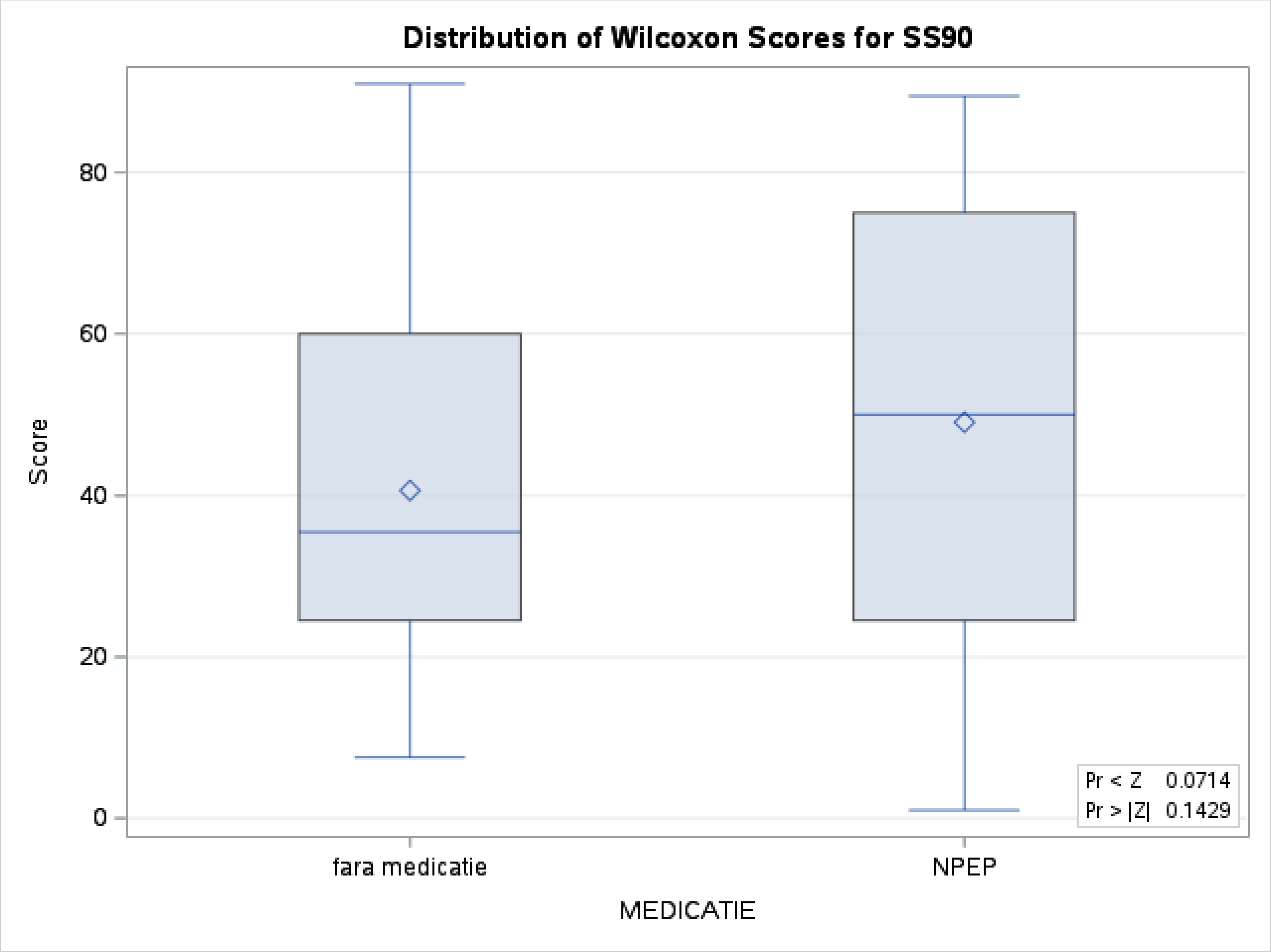

Variable: SS360 (SS360)
MEDICATIE = NPEP

Tests for Normality				
Test	Statistic		p Value	
Shapiro-Wilk	W	0.950761	Pr < W	0.0197
Kolmogorov-Smirnov	D	0.120686	Pr > D	0.0348
Cramer-von Mises	W-Sq	0.097838	Pr > W-Sq	0.1205
Anderson-Darling	A-Sq	0.605256	Pr > A-Sq	0.1125

Variable: SS360 (SS360)
MEDICATIE = fara medicatie

Tests for Normality				
Test	Statistic		p Value	
Shapiro-Wilk	W	0.948205	Pr < W	0.1179
Kolmogorov-Smirnov	D	0.188819	Pr > D	<0.0100
Cramer-von Mises	W-Sq	0.126619	Pr > W-Sq	0.0471
Anderson-Darling	A-Sq	0.732376	Pr > A-Sq	0.0506

Variable: SS360 (SS360)

MEDICATIE	Method	N	Mean	Std Dev	Std Err	Minimum	Maximum
NPEP		58	3.7586	4.3662	0.5733	-13.0000	14.0000
fara medicatie		33	0.7879	3.1400	0.5466	-6.0000	9.0000
Diff (1-2)	Pooled		2.9707	3.9692	0.8655		
Diff (1-2)	Satterthwaite		2.9707		0.7921		

MEDICATIE	Method	Mean	95% CL Mean		Std Dev	95% CL Std Dev	
NPEP		3.7586	2.6106	4.9066	4.3662	3.6912	5.3456
fara medicatie		0.7879	-0.3255	1.9013	3.1400	2.5252	4.1533
Diff (1-2)	Pooled	2.9707	1.2511	4.6904	3.9692	3.4620	4.6519
Diff (1-2)	Satterthwaite	2.9707	1.3955	4.5460			

Method	Variances	DF	t Value	Pr > |t|
Pooled	Equal	89	3.43	0.0009
Satterthwaite	Unequal	84.036	3.75	0.0003
Cochran	Unequal	.	3.75	0.0005

Equality of Variances				
Method	Num DF	Den DF	F Value	Pr > F
Folded F	57	32	1.93	0.0465

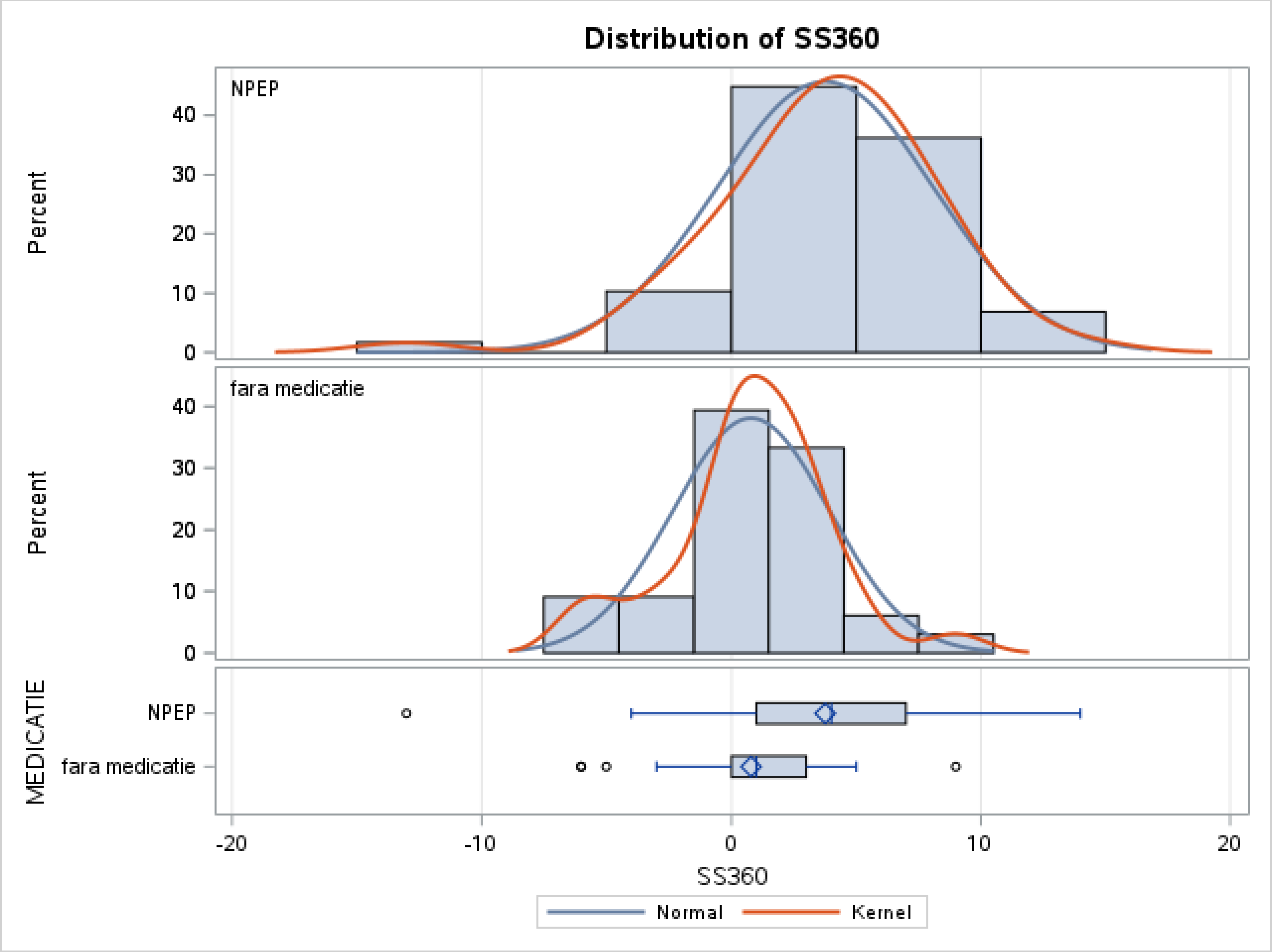

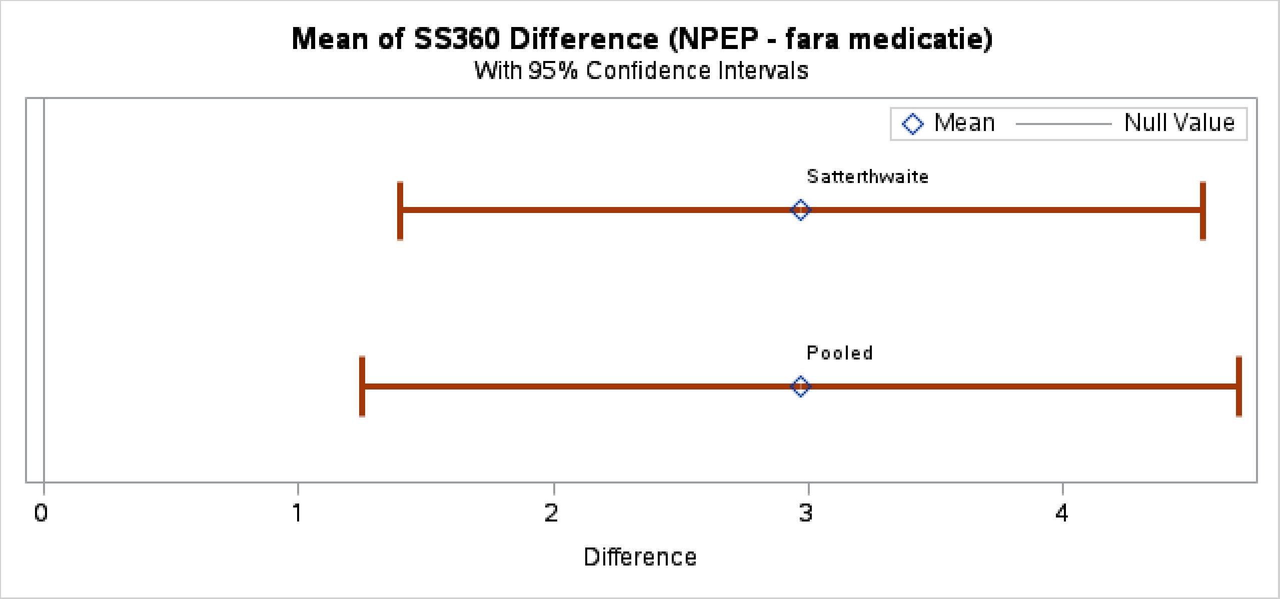

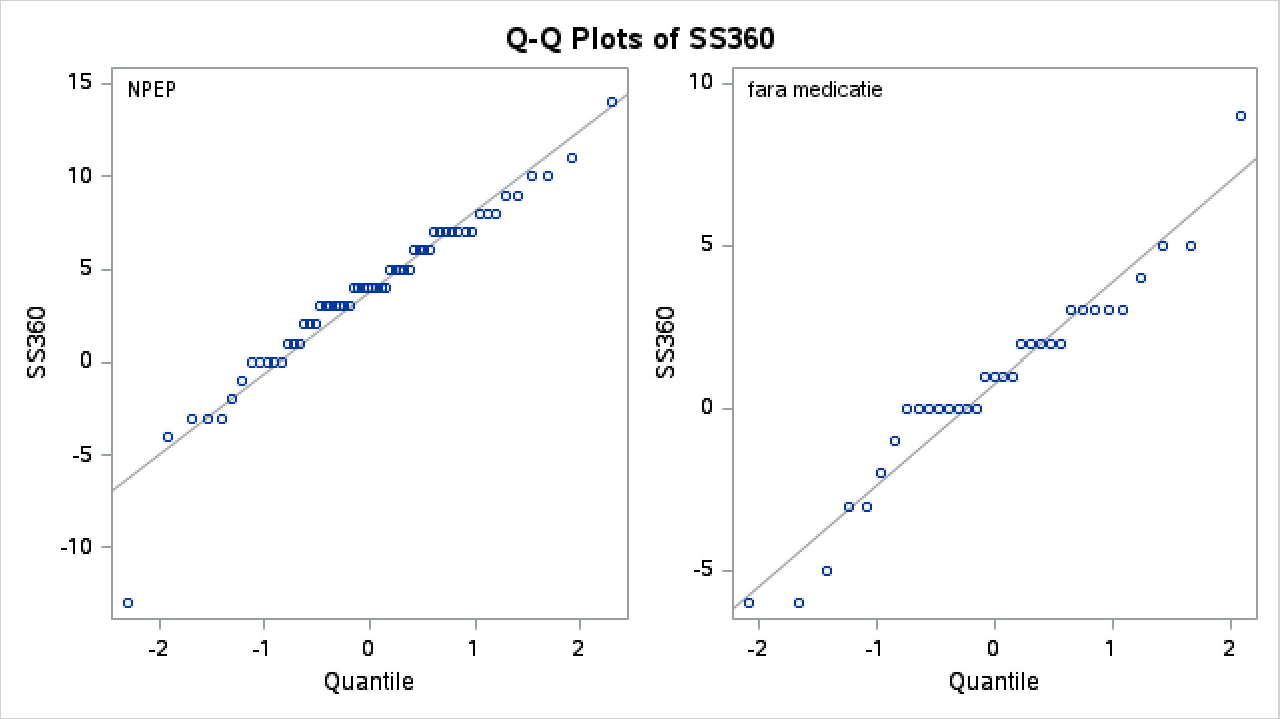

Wilcoxon Scores (Rank Sums) for Variable SS360 Classified by Variable MEDICATIE					
MEDICATIE	N	Sum of Scores	Expected Under H0	Std Dev Under H0	Mean Score
fara medicatie	33	1056.0	1518.0	120.622020	32.000000
NPEP	58	3130.0	2668.0	120.622020	53.965517
Average scores were used for ties.					

Wilcoxon Two-Sample Test					
				t Approximation	
Statistic	Z	Pr < Z	Pr > |Z|	Pr < Z	Pr > |Z|
1056.000	-3.8260	<.0001	0.0001	0.0001	0.0002
Z includes a continuity correction of 0.5.					

Kruskal-Wallis Test		
Chi-Square	DF	Pr > ChiSq

Kruskal-Wallis Test		
Chi-Square	DF	Pr > ChiSq
14.6700	1	0.0001

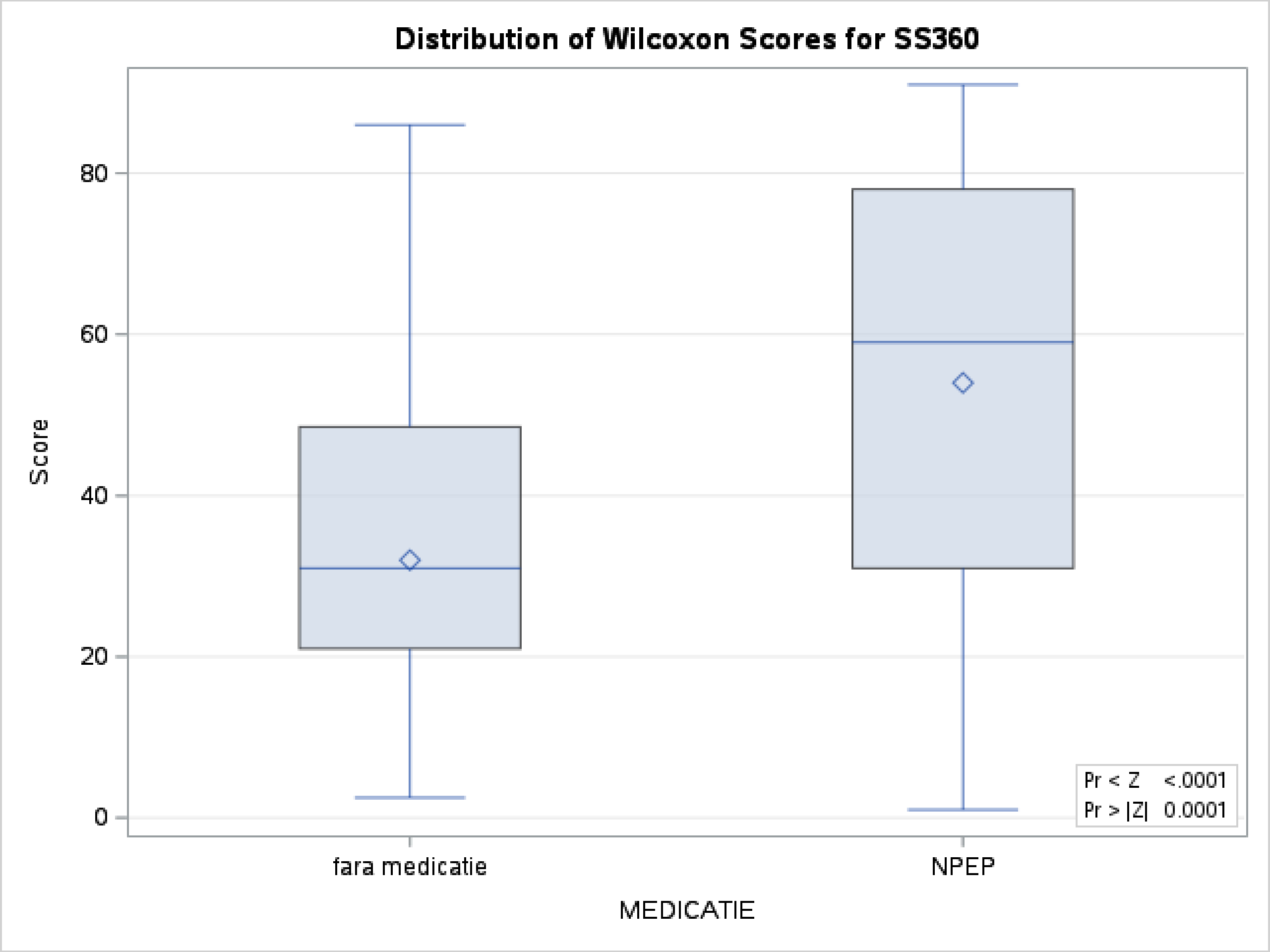

Variable: SSI90 (SSI90)
MEDICATIE = NPEP

Tests for Normality				
Test	Statistic		p Value	
Shapiro-Wilk	W	0.951676	Pr < W	0.0217
Kolmogorov-Smirnov	D	0.168418	Pr > D	<0.0100
Cramer-von Mises	W-Sq	0.255137	Pr > W-Sq	<0.0050
Anderson-Darling	A-Sq	1.295295	Pr > A-Sq	<0.0050

Variable: SSI90 (SSI90)
MEDICATIE = fara medicatie

Tests for Normality				
Test	Statistic		p Value	
Shapiro-Wilk	W	0.960469	Pr < W	0.2665
Kolmogorov-Smirnov	D	0.129998	Pr > D	>0.1500
Cramer-von Mises	W-Sq	0.102131	Pr > W-Sq	0.1012
Anderson-Darling	A-Sq	0.590591	Pr > A-Sq	0.1184

Variable: SSI90 (SSI90)

MEDICATIE	Method	N	Mean	Std Dev	Std Err	Minimum	Maximum
NPEP		58	-0.6897	2.1700	0.2849	-7.0000	5.0000
fara medicatie		33	-0.4848	1.6606	0.2891	-4.0000	3.0000
Diff (1-2)	Pooled		-0.2048	2.0018	0.4365		
Diff (1-2)	Satterthwaite		-0.2048		0.4059		

MEDICATIE	Method	Mean	95% CL Mean		Std Dev	95% CL Std Dev	
NPEP		-0.6897	-1.2602	-0.1191	2.1700	1.8346	2.6568
fara medicatie		-0.4848	-1.0737	0.1040	1.6606	1.3354	2.1965
Diff (1-2)	Pooled	-0.2048	-1.0721	0.6625	2.0018	1.7460	2.3462
Diff (1-2)	Satterthwaite	-0.2048	-1.0124	0.6028			

Method	Variances	DF	t Value	Pr > |t|
Pooled	Equal	89	-0.47	0.6401
Satterthwaite	Unequal	81.303	-0.50	0.6152
Cochran	Unequal	.	-0.50	0.6166

Equality of Variances				
Method	Num DF	Den DF	F Value	Pr > F
Folded F	57	32	1.71	0.1044
